# Prevalent symptoms and characteristics of the Long COVID-19 population: a scoping review*

**DOI:** 10.1590/1518-8345.7353.4479

**Published:** 2025-02-03

**Authors:** Karina Marques Prediger, Ana Cristina Ribeiro, Sílvia Carla da Silva André Uehara

**Affiliations:** 1 Universidade Federal de São Carlos, Departamento de Enfermagem, São Carlos, SP, Brazil; 2 Scholarship holder at the Fundação de Amparo à Pesquisa do Estado de São Paulo (FAPESP), Brazil

**Keywords:** Post-acute COVID-19 Syndrome, COVID-19, Signs and Symptoms, Population Characteristics, Pandemics, Review

## Abstract

to map the scientific literature on the clinical and demographic characteristics of Long COVID-19.

this is a scoping review based on the principles recommended by the JBI and the PRISMA guidelines for data extraction, carried out on four databases. The PCC strategy was used for data collection, and the results were described and diagrammed. The studies were selected after removing duplicates, individual and peer review.

an analysis of the 13 articles selected showed that Long COVID affects all age groups and people of both sexes, presenting a multiplicity of symptoms, such as fatigue (61.5%), dyspnea (46.1%), changes in smell and/or taste (38.6%), anxiety (15.3%) and cognitive impairment (30.7%). Females were found to be at increased risk of developing Long COVID.

identifying the symptoms prevalent in Long COVID contributes to public health strategies for diagnosing and assisting people affected by the disease. Future studies are recommended on the approach to the persistence of symptoms in Long COVID and the relationship between adherence to the vaccination schedule against COVID-19, gender, race/ethnicity, degree of susceptibility in the different age groups, level of education and income, as well as the most recurrent comorbidities in the population

## Highlights

**(1)** It was found that Long COVID affects all age groups of both sexes.

**(2)** Most common symptoms: fatigue, dyspnea and altered sense of smell and/or taste.

**(3)** Risk factors: female gender, COVID-19 severity and comorbidities.

## Introduction

The novel coronavirus disease (COVID-19) is not restricted to an acute illness, and about 10 to 50% of infected patients can progress to Long COVID, characterized by a multisystemic disease with heterogeneous symptoms^(1)^.

Long COVID can occur in individuals with probable or confirmed infection caused by the virus known as Severe Acute Respiratory Syndrome Coronavirus 2 (SARS-CoV-2)^(2)^. Long COVID is commonly observed three months after the diagnosis of infection in the acute phase and symptoms persist for at least two months and cannot be explained by an alternative diagnosis. In addition, symptoms may reappear after the initial recovery from an acute episode of COVID-19 or persist from the initial illness; furthermore, symptoms may fluctuate or recur between the period^(2)^.

In this scenario, COVID-19 can affect several organ systems and cause a series of symptoms that are not restricted to the lungs. Thus, the significant number of people who experience symptoms ranging from severe fatigue to neurological disorders experience the negative consequences of this phenomenon, while suffering damage to their quality of life^(3)^.

At the same time, Long COVID has a higher prevalence among comorbid patients who report fatigue, brain fog and myalgia as their main symptoms, which can be multiple and of varying intensity, depending on the area of the body affected. Furthermore, when a health professional sees a patient with these symptoms, they may associate them with psychosomatic causes such as depression and anxiety, which makes it difficult to diagnose Long COVID^(1)^.

In this way, the pathophysiological mechanisms of Long COVID are not yet fully understood and the diversity and complexity of the symptoms make it difficult to categorize them, which contributes to the gap in knowledge regarding the diagnosis and treatment of the disease. Therefore, studies on this subject are essential in order to contribute to the implementation of public health strategies and to help health services identify the main symptoms manifested and which people are more susceptible to developing Long COVID.

Therefore, treating the disease beyond its acute course requires research aimed at identifying the symptoms and pathophysiology of Long COVID, as well as ways of ensuring an accurate diagnosis in order to offer qualified and effective care. Therefore, this study aims to map the scientific literature on the clinical and demographic characteristics of Long COVID.

## Method

### Type of study

A Scoping Review (SR) was carried out based on the principles and phases recommended by the Joanna Briggs Institute (JBI), namely: (1) identification of the research question; (2) identification of relevant studies; (3) selection of studies; (4) data extraction; (5) separation, summarization and reporting of results; and (6) dissemination of results^(4)^. The protocol for this study is published in the Open Science Framework: https://doi.org/10.17605/OSF.IO/QRS4J.

### Scenario

The bibliographic research was conducted in the electronic databases: LILACS, PubMed, Scopus and Web of Science, and the searches were conducted using the following descriptors and/or alternative terms: acute post-COVID-19 syndrome, sequelae after SARS-CoV-2 infection, long-term COVID, clinical symptoms and signs and symptoms; as found in the Health Sciences Descriptors (DeCS) and Medical Subject Headings (MeSH) (Figure 1).

**Figure 1 -:** Search strategies used in the databases. São Carlos, SP, Brazil, 2023

**Database**	**Search strategy**
LILACS*	“Síndrome Pós-COVID-19^†^ Aguda” OR^‡^ “Afecções Pós-COVID” OR “COVID Longa” OR “Sequela Pós-Infecção por SARS-CoV-2^§^ Aguda” OR “Sequela Pós-Infecção por SARS-CoV-2 Aguda” OR “Post-Acute COVID-19 Syndrome” OR “Síndrome Post Agudo de COVID-19” OR “COVID de Longo Curso” AND^||^ “Sintomas Clínicos” OR “Sinais e Sintomas” OR “Sinais Clínicos” OR “Signs and Symptoms” OR “Signos y Síntomas”.
**PubMed** ^¶^	“Síndrome Pós-COVID-19 Aguda” OR “Afecções Pós-COVID” OR ” COVID Longa” OR “Sequela Pós-Infecção por SARS-CoV-2 Aguda” OR “Sequela Pós-Infecção por SARS-CoV-2 Aguda” OR “Post-Acute COVID-19 Syndrome” OR “Síndrome Post Agudo de COVID-19” OR “COVID de Longo Curso” NEAD/4^**^ “Sintomas Clínicos” OR “Sinais e Sintomas” OR “Sinais Clínicos” OR “Signs and Symptoms” OR “Signos y Síntomas”.
**Scopus**	“Síndrome Pós-COVID-19 Aguda” OR “Afecções Pós-COVID” OR ” COVID Longa” OR “Sequela Pós-Infecção por SARS-CoV-2 Aguda” OR “Sequela Pós-Infecção por SARS-CoV-2 Aguda” OR “Post-Acute COVID-19 Syndrome” OR “Síndrome Post Agudo de COVID-19” OR “COVID de Longo Curso” AND “Sintomas Clínicos” OR “Sinais e Sintomas” OR “Sinais Clínicos” OR “Signs and Symptoms” OR “Signos y Síntomas”.
**Web of Science**	“Síndrome Pós-COVID-19 Aguda” OR “Afecções Pós-COVID” OR “COVID Longa” OR “Sequela Pós-Infecção por SARS-CoV-2 Aguda” OR “Sequela Pós-Infecção por SARS-CoV-2 Aguda” OR “Post-Acute COVID-19 Syndrome” OR “Síndrome Post Agudo de COVID-19” OR “COVID de Longo Curso” AND “Sintomas Clínicos” OR “Sinais e Sintomas” OR “Sinais Clínicos” OR “Signs and Symptoms” OR “Signos y Síntomas”.

*LILACS = Latin American and Caribbean Health Sciences Literature;^†^Post-COVID-19 = Post Corona Virus Disease 2019;^‡^OR = Or; ^§^SARS-CoV-2 = Severe Acute Respiratory Syndrome Coronavirus 2; ^||^AND = E; ^¶^PubMed = Public MedLars; **NEAD/4 = Proximity in up to 4 terms

### Period

Data searches took place between May and June 2023.

### Selection criteria

The inclusion criteria were: original articles, published between January 2020 and January 2023 and in Portuguese, English and/or Spanish. Articles that were not available in full, whose titles and abstracts did not answer the guiding question, as well as opinion articles, editorials, reviews and book chapters were excluded. The study period refers to the beginning of the COVID-19 pandemic and consequently of publications related to the persistence of symptoms of this disease.

### Data collection

In order to conduct this study, the PCC (Population, Concept and Context) strategy was used, with “P” being people with Long COVID; “C” being symptoms and “C” being the COVID-19 pandemic, and the following guiding question was defined: “What symptoms of Long COVID are presented by people, considering the clinical and demographic characteristics?”.

### Instruments used to collect information

To select the studies, after implementing the search strategy in the aforementioned electronic databases, the articles were imported into the StArt (State of the Art through Systematic Review) web application, in order to select the studies on two levels. This review tool was developed by the Software Engineering Research Laboratory (LaPES) at the Federal University of São Carlos (UFSCar)^(5)^. Thus, during the first stage of selection, the titles and abstracts were read, followed by the full references. The reference lists of all the studies found were also checked.

### Data processing and analysis

The eligible studies were retrieved in their entirety and assessed by three researchers, and any differences were discussed until a consensus was reached and the final selection made. PRISMA (extension for scoping reviews) guidelines were followed for data extraction and presentation^(6)^. The process of selecting the studies and the relevant information extracted from each selected article are presented in figures, in descriptive format.

### Ethical aspects

As this is a scoping study, approval by the Research Ethics Committee is not required.

## Results

**Figure 2 -:**
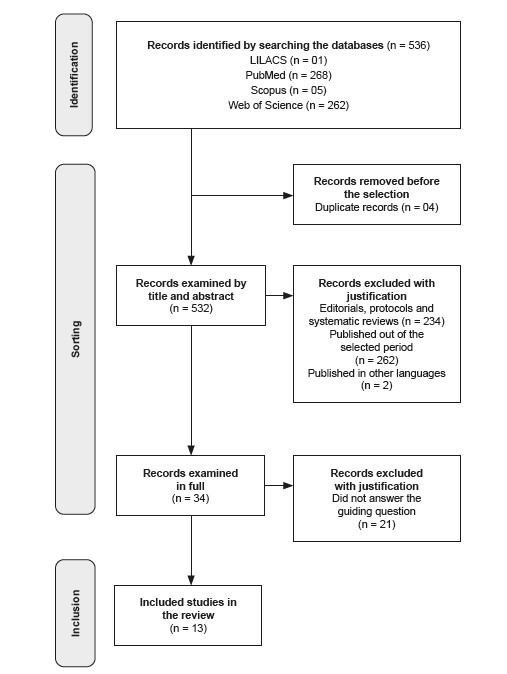
Flow diagram of the article selection process, PRISMA-ScR. São Carlos, SP, Brazil, 2023

A total of 536 articles were imported into StArt, of which four were excluded for being duplicates. Thus, 532 articles were considered for title and abstract analysis, of which 498 studies were excluded because they did not meet the inclusion criteria. Thus, 34 articles were included for full reading. After reading, 21 studies were excluded because they did not answer the guiding question, and 13 studies were selected in the end (Figure 2).

In terms of the types of studies included in this review, Figure 3 shows that 8 (61.5%) were cohort studies, 3 (23.0%) were case studies, 1 (7.6%) was a case-control study and 1 (7.6%) was a descriptive study. As for the countries of the studies, 2 (15%) were carried out in the United States; in addition, countries such as Saudi Arabia, Belgium, Brazil, China, Egypt, Spain, France, Greece, Italy, London and Switzerland appear with only 1 (7.6%) study each.

**Figure 3 -:** Description of articles according to author, year, location, objective, type of study, sample and main results. São Carlos, SP, Brazil, 2023

**Author, year and country**	**Objective**	**Type of study and sample (n)**	**Main results**
Eita AAB, 2021, United States of America^(7)^	Presenting a case of post-acute COVID-19 syndrome*, showing parostomia, dysgeusia and a white-coated fatty tongue with prominent papillae	Case study. n = 1.	After 14 days of COVID-19* infection, a 31-year-old female patient complained of dysgeusia; she had a greasy, unpleasant white tongue and a distorted sense of smell. The symptoms improved over four weeks with drug treatment and after viral elimination.
Judant EM, 2021, France^(8)^	To determine the characteristics of patients with respiratory complaints and the relationships between dyspnoea, radiological abnormalities and functional impairment	Cohort study. n = 478 (case) and n =177 (control).	Individuals hospitalised for COVID-19* were assessed by telephone consultation. The average age of the sample was 61 and 42.1 per cent were female. 4 months after the diagnosis of the viral infection, 78 people reported dyspnoea and 23 reported coughing. Compared to individuals without dyspnoea, those with dyspnoea were younger (56.1±12.3 *versus* 61.9±16.6 years, ^p†^=0.001). Individuals with dyspnoea had more severe initial episodes of COVID-19*, longer hospital stays [13 (7-23) *versus* 8 (4-14) days, p<0.001] and more frequent admissions to the Intensive Care Unit (56.4 *versus* 24.5%, p<0.001).
Moreno-Perez O, 2021, Spain^(9)^	Analysing the incidence of post-acute COVID-19 syndrome* and its components and assessing the risk factors associated with the acute phase of infection	Cohort study. n = 277.	Individuals with COVID-19* were assessed 77 days after infection. The average age was 62 years and 52.7 per cent were male. Post-COVID-19* symptoms were detected in 50.9 per cent of patients. The most frequent symptoms were dyspnoea and fatigue. Anosmia-dysgeusia was associated with younger age (<65 years 24.9% (48/194) vs >65 years 13.5% (11/83). Relevant neurological symptoms (headache, memory disorders/cognitive deterioration or both) were present in 33 patients (11.9%). After multivariate adjustment, no baseline clinical characteristics, intensive care unit admission, length of hospital stay or treatment behaved as independent predictors of post-COVID-19 syndrome*.
Penetra SLS, 2021, Brazil^(2022)^	To describe a case of prolonged COVID-19* after SARS-CoV-2 Gamma infection one year after the first infection in a healthcare worker vaccinated with CoronaVac in Brazil	Case study. n = 1.	A 43-year-old male patient with no comorbidities. When infected with COVID-19*, he presented with fever, headache, rhinorrhoea and dry cough; symptoms disappeared within 14 days. Nine months after infection, she received the first dose of the vaccine and 28 days after the second dose. 67 days after the second dose, he developed a new episode of the COVID-19* Gamma strain. At the time, he had the same symptoms; however, they persisted for 18 weeks after the acute infection with the exception of headache and blurred vision. Both infections did not require hospitalisation.
Tleyjeh IM, 2021, Saudi Arabia^(11)^	To evaluate the frequency of new or persistent symptoms in COVID-19* patients after hospital discharge and identify associated risk factors	Cohort study. n = 222.	Follow-up telephone interviews were conducted with individuals with COVID-19* after a median of 122 days after hospital discharge. The majority of patients were men (77%) with a mean age of 52.47 (± 13.95) years. The time to diagnosis of post-COVID-19* symptoms ranged from 21 days to 4 months after discharge. 56.3 per cent of patients complained of symptoms persisting; 66 (29.7 per cent) experienced them for >21 days and 64 (28.8 per cent) reported not having returned to their initial health. Shortness of breath (40.1%), cough (27.5%) and fatigue (29.7%) were the most frequently reported symptoms. After multivariate adjustments, female gender, pre-existing hypertension and length of hospital stay were associated with an increased risk of new or persistent symptoms. Age, pre-existing lung disease and emergency room visits increased the likelihood of not recovering completely from acute COVID-19*.
Tohamy D, 2021, Egypt^(12)^	Evaluating the ocular manifestations of post-acute COVID-19 syndrome*	Cohort study. n = 100 (case) and n = 100 (control).	The time to diagnosis of post-COVID-19* conditions ranged from 1 to 3 months. The individuals in the sample had no history of COVID-19* vaccination. The average age was 55.5 years in the COVID group versus 56.5 years in the control group. In the COVID group, 57 per cent of the patients were male versus 51 per cent in the control group. In the COVID group, 5 patients had retinal vascular occlusion, 2 patients had anterior ischaemic optic neuropathy, 3 patients had uveitis and 2 patients had central serous chorioretinopathy. In the control group, 2 patients had retinal vascular occlusion and none had uveitis or central serous chorioretinopathy.
Carter SJ, 2022, United States of America^(13)^	To describe the concomitant effects of SARS-CoV-2 infection on functional status, mood and leisure-time physical activity in post-acute COVID-19 syndrome*	Case-control study. n =17 (case) and n =15 (control).	The diagnosis time for post-COVID-19* conditions was approximately 4 weeks. 32 women were included, 17 diagnosed with COVID-19* and 15 controls. Diastolic blood pressure (*p* = 0.031, ES = 0.80) and RPP^‡^ (*p* = 0.039, ES = 0.75) were significantly higher among participants in the COVID-19* group. In the COVID-19* group, the following symptoms were observed: 41 per cent with joint/muscle pain for around 300 days, 53% with cough over the same period; 29 per cent with fatigue, 12 per cent with cognitive impairment and 71 per cent with loss of taste/smell over a period of approximately 100 days, as well as 35 per cent with shortness of breath.
Fanous J, 2022, London^(14)^	To objectively assess neuromuscular fatigability concomitant with fatigue and cognitive dysfunction related to COVID-19* 12 months after infection	Case study. n = 1.	The diagnosis time for the post-COVID-19* conditions was around 12 months. A 27-year-old male patient with ongoing post-COVID-19* complaints of fatigue and cognitive dysfunction, anosmia and ageusia, severe mental confusion, loss of appetite and insomnia for more than a year after infection.
Jamoulle M, 2022, Belgium^(1)^	To provide a qualitative and quantitative description of the state of health of patients with Post-Acute COVID-19 Syndrome*.	Cohort study. n = 55.	The time to diagnosis of post-COVID-19* conditions ranged from 3 to 27 months. 12% of the sample population had not been vaccinated during the study period. Among the patients, 72.7 per cent were women, aged between 12 and 79 years. After one year of follow-up, three types of clinical evolution can be distinguished from the onset of acute COVID-19* in 52 of the 55 patients: Grade 1 (mild) - duration of 3 to 8 months, respiratory impairment, fatigue, sternal pain and exhaustion on exertion (9 women and 7 men); Grade 2 (severe) - duration of 6 to 18 months, extreme fatigue, exhaustion on exertion, anosmia, cognitive disturbance, mnesic disturbance (12 women and 3 men); Grade 3 (very severe) - lasting from 12 to 27 months, constant exhaustion, impossible efforts, useless cognitive revalidation, hypersomnia, weight gain due to inactivity, severe and persistent memory disturbances and considerable anxiety. Other variable symptoms include brain fog with memory impairment and word retrieval deficit, dyspnoea on exertion and incapacitating state.
Kim Y, 2022, South Korea^(15)^	To identify prevalent symptoms after 12 months of COVID-19 infection* in recovered patients, factors associated with neurological symptoms and the long-term impact of COVID-19 on quality of life	Cohort study. n = 900 (case) and n = 241 (control)	The time taken to diagnose post-COVID-19* conditions ranged from 6 to 12 months. The average age of respondents was 37 years and 164 were women. Symptoms persisting for more than 12 months were higher in those classified as moderate or greater disease severity during acute COVID-19* infection. The most frequent symptom lasting up to 12 months was difficulty concentrating, observed in 54 people, followed by cognitive dysfunction (51), amnesia (48), depression (43) and fatigue and anxiety (39). Among the persistent symptoms related to COVID-19*, psychiatric problems had a greater impact on quality of life than any other symptoms. Older age, female gender and disease severity were identified as risk factors for persistent neuropsychiatric symptoms.
Liu Q, 2022, China^(16)^	To investigate whether the composition of the intestinal microbiota is linked to post-acute syndrome	Cohort study. n = 106 (case) and n = 68 (control)	The time to diagnosis of post-COVID-19* conditions ranged from 3 to 6 months. Patients with COVID-19* exacerbations were followed up from admission to six months. The average age of the COVID group was 48.3 years and 52.9 per cent were female. The following comorbidities were found: 17% hypertensive and 15.1% with diabetes mellitus. 81.1% of patients reported post-acute COVID-19 syndrome* within 3 months. The most common symptoms were: fatigue (31.3%), poor memory (28.3%), hair loss (21.7%), anxiety (20.8%) and difficulty sleeping (20.8%). There were no significant differences in age, gender, comorbidities, medication use and COVID-19* severity in patients with or without COVID-19* post-acute syndrome.
Mazzitelli M, 2022, Italy^(17)^	To assess the prevalence and factors associated with severe COVID-19* and post-acute COVID-19* syndrome	Cohort study. n = 123.	The diagnosis time for post-COVID-19* conditions was 6 months. Patients diagnosed with HIV^§^ and concomitantly with COVID-19* were analysed. Of these, 79.7 per cent were male, 77.2 per cent were Caucasian and the average age was 51 years. None of the patients had been vaccinated for COVID-19*. Regarding comorbidities: 30.9 per cent were hypertensive, 17.9 per cent diabetic and 15.4 per cent were overweight/obese; among the 75 patients who had symptomatic COVID-19* infection and survived, 26.7 per cent reported post-acute COVID-19* syndrome. As for symptoms: 80% reported asthenia; 50% exertion-related shortness of breath; 25% recurrent headache; 15% significant hair loss; 10% loss of sense of smell; 0.5% chronic diarrhoea; and 2 patients reported cardiovascular complications (acute myocardial infarction and rhythm changes).
Zachariou A, 2022, Greece^(18)^	To evaluate the consequences on bladder function in patients with post-acute COVID-19 syndrome* transferred to long-term care inpatient rehabilitation after initial treatment for COVID-19 pathophysiology*	Descriptive study. n = 147.	The time to diagnosis of post-COVID-19* conditions ranged from 4 to 7 weeks. A study of patients with post-COVID-19 syndrome* referred to the rehabilitation centre for long-term care. Of these, only 106 agreed to take part in the study, 45 (43.4%) female and 60 (56.6%) male. The average age of the patients was 59.5. We identified 75 patients with symptoms associated with overactive bladder (OAB) between 4 and 7 weeks after presenting COVID-19*. Of these, 9 patients refused to answer the questionnaires; 44 patients had newly diagnosed overactive bladder and 22 patients had worsening symptoms of overactive bladder. All the patients complained of nocturia.

*COVID-19 = Corona Virus Disease 2019*;* ^†^p = Significance level; ^‡^RPP = Rate-Pressure Product; ^§^HIV = Human Immuno-Deficiency Virus

With regard to demographic data, it was found that in 5 (38.4%) studies the average age of Long COVID cases ranged from 42.4 to 55.5 years, in studies where the sample was made up of both sexes, four described their samples with the highest percentages corresponding to men and three studies were predominantly female.

The most frequently observed symptoms were fatigue (61.5%)^(1,7,9,11,13-16)^, dyspnoea (46.1%)^(1,8-9,11,13,17)^, changes in smell and/or taste (38.6)^(1,7,9,13,17)^, anxiety (15.3%)^(1,15)^ and cognitive impairment (30.7%)^(1,13-14,16)^.

With regard to the clinical characteristics of the presence of pre-existing illnesses in people who manifested signs and symptoms suggestive of Long COVID-19, only 5 (38.4%) studies described the most frequent comorbidities observed in their samples, with diabetes mellitus and hypertension being predominantly reported^(9,11,15-17)^.

A clinical variable that was little addressed in the studies was the relationship between the COVID-19 vaccination programme and the development of Long COVID. 9 (69.24%) studies did not mention vaccination status^(7-9,11,13-16,18)^, 2 (15.3%) stated that the study population had not been vaccinated^(12,17)^ and 1 (7.6%) showed that around 12% of the population investigated had not been vaccinated during the study period^(1)^. None of the studies analysed the association between vaccination and the development of Long COVID symptoms.

Other gaps identified in the selected studies refer to the lack of analysis of the association of Long COVID with demographic variables such as education and income^(1,7-18)^ or clinical variables on the history of hospitalisation for COVID-19^(1,7,12-14,18)^.

With regard to the risk factors associated with the development of Long COVID, only two studies have described a positive association related to the severity of the acute phase of COVID-19 disease, the presence of comorbidities, longer length of stay and hospitalisation in the Intensive Care Unit, older age and female gender with an increased risk of manifesting some symptoms of Long COVID^(8,11,15)^. On the other hand, one study found that the presence of anosmia-dysgeusia was associated with younger age (under 65)^(9)^.

## Discussion

Analysing the studies selected in this review identified a wide range of symptoms reported in people who developed Long COVID, among the most frequent being fatigue, dyspnoea, changes in smell and taste and cognitive disorders related to memory. In addition, it was pointed out that diseases such as hypertension and diabetes were commonly found comorbidities in people. In this analysis, a higher prevalence of Long COVID-19 was observed in the samples of people corresponding to the adult age group and in both sexes. However, some studies have associated the female sex as a risk factor for developing the disease, other factors associated with an increased risk of developing Long COVID were related to the severity of the COVID-19 infection, longer hospitalisation and older age. Also, in relation to the vaccination scheme against COVID-19, it was observed that this topic was not addressed in most of the studies analysed on the manifestation of symptoms in Long COVID disease.

It should be noted that in the first year of the pandemic, in 2020, the term “Long COVID’ was used to describe patients who had recovered from the disease, but who still had lasting effects from the infection for longer than expected^(19)^. In addition, the degree of COVID-19 infection may be related to different symptoms manifested in the development of Long COVID. Thus, Long COVID was observed more frequently in people who developed the severe form of COVID-19 compared to less severe cases^(20)^. In addition, the risk of developing Long COVID increases with age, but it can affect the entire population, including children^(21-22)^.

Although the pathophysiology of Long COVID is not fully understood, hypotheses explaining the disease may include the presence of persistent reservoirs of SARS-CoV-2 in tissues, immune dysregulation, the impact of the virus on the microbiota, autoimmunity, preparation of the immune system based on molecular mimicry, microvascular blood coagulation with endothelial dysfunction and dysfunctional signalling in the brainstem and/or vagus nerve^(22-23)^.

Regarding the symptoms manifested in Long COVID, it has been described that these can be superimposed on various conditions that are not related to the COVID-19 infection itself, such as, for example, a post-intensive care syndrome in cases that required hospitalisation or even the exacerbation of pre-existing health conditions^(24-25)^.

In this context, the symptoms manifested in Long COVID can have different aetiologies. Thus, in the approach to long-term respiratory symptoms, these may be caused by pulmonary vascular disorders resulting from pulmonary vascular damage in the microvessels suffered in the acute infection of the SARS-CoV-2 virus. One of the most commonly reported symptoms, dyspnoea, in the absence of lung damage, could be related to inadequate regulation of ventilation resulting from disorders of the autonomic nervous system, with potential damage to intrathoracic reflex receptors or the brainstem/cortical brain zones^(23,26)^. As for the manifestation of pain, including joint and muscle pain, it could be attributed to thromboinflammatory mechanisms in relation to tissue damage and autoimmune processes^(27-28)^.

As for the neurological symptoms presented in Long COVID, an analysis pointed out that persistent sequelae could be related to acute neurological complications in the SARS-CoV-2 virus infection, resulting from brain damage or other factors related to hospitalisation. In this way, the manifestation of cognitive disorders, headaches and changes in smell and/or taste could depend on a pathophysiology independent of the acute phase. One explanation could be that the spread of the SARS-CoV-2 virus in the brain, via the nasal cavity or the bloodstream, results in neuroinflammation, which, if persistent, could be responsible for neurocognitive impairment or mental health disorders^(23)^.

Thus, clinical examinations of people with Long COVID show a decrease in serotonin caused by COVID-19 infection due to reduced tryptophan absorption, thrombocytopenia and increased expression of flavoenzyme monoamine oxidase, which results in decreased vagal and hippocampal activation and also considerable cognitive impairment. It has also been found that reduced serotonin and vagus nerve dysfunction can be associated with Long COVID, as they are suggestive of a pathway subsequent to viral infection^(29)^.

However, based on the idea that the metabolomic, proteomic and immunological phenotyping of patients who contracted SARS-CoV-2 combined with a diversity of clinical symptoms with potential biomarkers for COVID-19, deregulated metabolism and inflammation may contribute to the symptoms of Long COVID. In this context, it is noteworthy that total blood triglycerides and the metabolites lactate and pyruvate were higher and lipoproteins lower in patients with Long COVID when compared to healthy controls^(30)^.

Furthermore, it was possible to verify that cytokines or female and/or male gender were related to metabolites such as citrate, glutamate and histidine in patients who presented symptoms such as chronic fatigue, dyspnoea and mental confusion during the long course of the disease. Finally, various cytokines and chemokines have been correlated with metabolites and lipoproteins, with metabolic dysregulation and inflammation being considered potential factors in the development of Long COVID^(30)^.

Among the risk factors for developing Long COVID-19, hospitalisation as a result of the infection, increasing age, being female and having comorbidities have been described^(31)^. In this context, it is known that diseases commonly found in the population such as diabetes, when poorly controlled, cause organ damage, specifically causing microvascular lesions that can be exacerbated in the severe form of SARS-CoV-2 infection. In addition, the pre-existing inflammatory state can remain exacerbated and worsen after infection^(32)^.

In addition, acute coronary syndromes, heart failure, arrhythmias, stroke and thromboembolism persist beyond the initial stage of SARS-CoV-2 infection, lasting for several months. Cardiopulmonary symptoms such as chest pain, shortness of breath, fatigue and postural orthostatic tachycardia can be common in Long COVID and associated with significant disability and increased anxiety. However, the pathophysiological mechanisms for late cardiovascular complications are not well understood^(33)^.

The literature points to divergences in relation to the prevalence of Long COVID, a study that analysed the population of the northern Netherlands described that the disease can occur in around 1 in 8 people with the disease in the general population, while another study in Italy reported that 9 out of 10 patients after recovering from the disease still had at least one symptom 60 days after onset^(19,34)^.

In addition, the prevalence of Long COVID differs between men and women, considering the physiological and hormonal differences that influence the immune system. Thus, women have higher immunoglobulin levels, causing them to develop stronger immune responses after immunisation or infection compared to men, while they are more susceptible to autoimmune diseases^(35)^.

Thus, women can present different risk factors for Long COVID depending on their hormonal status. In this context, menopause is a phenomenon that is associated with an increased risk of the disease, with postmenopausal women having a higher incidence of COVID-19 when compared to premenopausal women. This suggests that oestrogen may be associated with the severity of the disease; it also highlights that ACE2 is found on the X chromosome, with oestrogen being a negative regulator of ACE2 expression. Furthermore, Toll-like receptor 7 (TLR7), a regulator of interferon (IFN) production, is considered to be an immune gene on the X chromosome that causes greater IFN signalling in COVID-19 and better viral clearance in women; however, continuous signalling predisposes them to a greater risk of developing Long COVID^(36)^.

However, in both sexes, the clinical findings, alterations in T cells, the reduced number of cluster of differentiation 4+ (CD4+) and cluster of differentiation 8+ (CD8+) effector memory cells and the elevated expression of the PD1 protein in central memory cells were found to persist for at least 13 months in individuals with Long COVID who had mild COVID-19^(22,37)^.

In individuals who developed Long COVID, a decrease in immunoglobulin M (IgM) was detected from primary infection to 6-month follow-up. Furthermore, while immunoglobulin G1 (IgG1) remained unchanged, immunoglobulin G3 (IgG3) was lower in patients with Long COVID, the opposite occurring in IgG3 concentrations in mild and severe cases of COVID-19. Thus, it was possible to observe an immunoglobulin signature that, combined with age, history of bronchial asthma and five symptoms during the primary infection, can predict the risk of Long COVID regardless of the period in which the blood was taken^(38)^.

Using SARS-CoV-2 specific immunoglobulin G (IgG) titers distributed equally over a period according to age, gender and absence/presence of long-lasting symptoms, patients were vaccinated between the 10th and 15th month after infection, identifying that early vaccination is more effective than late vaccination. In addition, vaccination administered before SARS-CoV-2 infections is associated with a lower prevalence of persistent symptoms^(39)^.

In this context, although vaccination against COVID-19 was a clinical variable not analyzed in the studies selected in this review, its relationship with Long COVID deserves to be highlighted. The literature indicates that the double vaccination culminated in a temporary beneficial effect on long-lasting symptoms lasting between 21 and 67 days^(39)^. A systematic review that analyzed data on vaccination before and after infection with the SARS-CoV-2 virus, in relation to vaccination against COVID-19 before infection, showed that individuals had a significant reduction in the incidence of Long COVID. On the other hand, when analyzing remission and recovery from Long COVID, there are chances of non-recovery when patients were vaccinated after infection^(40)^.

Thus, vaccination against COVID-19, in addition to reducing the severity of the disease and hospitalization rates, has been associated with a considerable reduction in the development of symptoms of Long COVID^(40-41)^. From this perspective, the importance of adherence to vaccination against COVID-19 is noteworthy since, if it is recognized, it acts as an instrument of protection against the development of the long course of the disease, given that there are still no widely effective treatments for both the acute disease and Long COVID^(22)^.

Finally, it is important to note that health care for people with Long COVID can be compromised, especially in low- and middle-income countries, due to limited health systems with few resources, which can be a great demand on health systems that are already overloaded and further exacerbate health inequalities^(42)^.

This scoping review helps to identify the most frequent symptoms of Long COVID, including fatigue, dyspnea, changes in smell and taste and cognitive disorders related to memory. In addition, the selected studies showed a higher prevalence of Long COVID in people in the adult age group and in both sexes.

Limitations of the study include the inclusion of only studies in Portuguese, English and Spanish, articles that were available in full text and indexing databases not included in this research.

## Conclusion

The mapping of the literature showed the diversity of symptoms in people who developed Long COVID, being more prevalent in adults, of both sexes and with the presence of comorbidities. Due to the high burden of persistent symptoms caused by SARS-CoV-2 infection, Long COVID is a challenge for health services, especially with regard to the diversity of symptomatic manifestations. This situation implies difficulties for the quality of life of people affected by Long COVID.

This review found important gaps that can influence the understanding of Long COVID, so future studies are recommended on the approach to the severity and persistence of symptoms presented in Long COVID and the relationship between adherence to the vaccination schedule against COVID-19, gender, race/ethnicity, degree of susceptibility in the different age groups, level of education and income, in addition to the most recurrent comorbidities in the population. Further studies using these variables could contribute to the effectiveness of public health strategies for diagnosing and assisting people affected by the disease.
