# Supplementary material for: Prevalent symptoms and characteristics of the Long COVID-19 population: a scoping review
Source: Rev Lat Am Enfermagem. 2025 Feb 3;33:e4479. doi: 10.1590/1518-8345.7353.4479 (PMC11789739; doi:10.1590/1518-8345.7353.4479)
Supplement: Translation

# Translation: Síntomas prevalentes y características de la población en la COVID prolongada: una revisión de alcance*

**DOI:** 10.1590/1518-8345.7353.4478

**Authors:** Karina Marques Prediger, Ana Cristina Ribeiro, Sílvia Carla da Silva André Uehara

**Affiliations:** 1 Universidade Federal de São Carlos, Departamento de Enfermagem, São Carlos, SP, Brasil; 2 Becaria de la Fundação de Amparo à Pesquisa do Estado de São Paulo (FAPESP), Brasil

**Keywords:** Síndrome Post Agudo de COVID-19, COVID-19, Signos y Síntomas, Características de la Población, Pandemias, Revisión

## Abstract

mapear la literatura científica sobre las características clínicas y demográficas de la COVID prolongada.

se trata de una revisión de alcance basada en los principios recomendados por el JBI y las directrices del PRISMA para la extracción de datos, realizada a partir de cuatro bases de datos. Se utilizó la estrategia PCC para la recolección de datos, siendo los resultados descritos y diagramados. La selección de los estudios se realizó después de la eliminación de duplicados, evaluación individual y en pares.

un análisis de los 13 artículos seleccionados mostró que la COVID prolongada afecta a todas las franjas etarias y a personas de ambos sexos, presentando una multiplicidad de síntomas, como fatiga (61,5%), disnea (46,1%), alteraciones en el olfato y/o gusto (38,6%), ansiedad (15,3%) y deterioro cognitivo (30,7%). En el sexo femenino se identificó un mayor riesgo de desarrollar COVID prolongada.

la identificación de los síntomas prevalentes en la COVID prolongada contribuye a la estrategia de salud pública, para el diagnóstico y asistencia a las personas afectadas por la enfermedad. Se recomiendan estudios futuros sobre el abordaje de la persistencia de los síntomas en la COVID prolongada y la relación de la adherencia al esquema de vacunación contra la COVID-19, sexo, raza/etnia, grado de susceptibilidad en las diferentes franjas etarias, nivel educativo e ingresos, además de las comorbilidades más frecuentes en la población.

## Destacados

**(1)** Se identificó que la COVID prolongada afecta a todas las franjas etarias de ambos sexos.

**(2)** Síntomas más frecuentes: fatiga, disnea y alteración en el olfato y/o gusto.

**(3)** Factores de riesgo: sexo femenino, gravedad de la COVID-19 y comorbilidades.

## Introducción

La enfermedad del nuevo coronavirus (COVID-19) no se restringe solo a una enfermedad aguda, ya que alrededor del 10 al 50% de los pacientes infectados pueden desarrollar COVID prolongada, caracterizada por ser una enfermedad multisistémica con síntomas heterogéneos^(1)^. La COVID prolongada puede ocurrir en individuos con infección probable o confirmada ocasionada por el virus denominado *Severe Acute Respiratory Syndrome Coronavirus* 2 (SARS-CoV-2)^(2)^.

La COVID prolongada se observa comúnmente después de tres meses del diagnóstico de la infección en la fase aguda, y los síntomas persisten por al menos dos meses, sin poder ser explicados por un diagnóstico alternativo. Además, los síntomas pueden reaparecer tras la recuperación inicial de un episodio agudo de COVID-19 o persistir desde la enfermedad inicial; asimismo, los síntomas pueden fluctuar o recurrir durante este período^(2)^.

En este contexto, la COVID-19 puede afectar varios sistemas orgánicos y ocasionar una serie de síntomas que no se limitan solo a la región pulmonar. De este modo, el número significativo de personas que presentan síntomas que van desde fatiga severa hasta trastornos neurológicos experimentan las consecuencias negativas de este fenómeno, a la vez que sufren deterioros en su calidad de vida^(3)^.

Concomitantemente, la COVID prolongada presenta una mayor prevalencia entre pacientes con comorbilidades que reportan fatiga, niebla mental y mialgia como principales síntomas, los cuales pueden ser múltiples y de intensidad variable, dependiendo de la zona del cuerpo afectada. Además, el profesional de salud que atiende a un paciente con estos síntomas puede asociarlos a causas psicosomáticas como depresión y ansiedad, lo que dificulta el diagnóstico de COVID prolongada^(1)^.

De esta manera, los mecanismos fisiopatológicos de la COVID prolongada aún no están totalmente esclarecidos, y la diversidad y complejidad de los síntomas dificultan su categorización, lo que contribuye a la falta de conocimiento en cuanto al diagnóstico y tratamiento de la enfermedad. Por tanto, son esenciales estudios que aborden esta temática para contribuir a la implementación de estrategias de salud pública y apoyar a los servicios de salud en la identificación de los principales síntomas manifestados y de las personas más susceptibles al desarrollo de COVID prolongada.

Por lo tanto, tratar la enfermedad más allá de su curso agudo requiere investigaciones que busquen identificar la sintomatología y la fisiopatología de la COVID prolongada, así como formas de asegurar un diagnóstico preciso con el fin de ofrecer una atención cualificada y efectiva. De este modo, este estudio tiene como objetivo mapear la literatura científica sobre las características clínicas y demográficas de la COVID prolongada.

## Método

### Tipo del estudio

Se realizó una *Scoping Review* (SR) basada en los principios y fases recomendados por el *Joanna Briggs Institute* (JBI), a saber: (1) identificación de la cuestión de investigación; (2) identificación de estudios relevantes; (3) selección de los estudios; (4) extracción de datos; (5) separación, resumen e informe de resultados; y (6) divulgación de los resultados(4). El protocolo de este estudio está publicado en *Open Science Framework*: https://doi.org/10.17605/OSF.IO/QRS4J.

### Escenario

La investigación bibliográfica se llevó a cabo en las bases de datos electrónicas: LILACS, PubMed, Scopus y *Web of Science*, y las búsquedas se realizaron utilizando los descriptores y/o términos alternativos: síndrome post-COVID-19 aguda, secuela post-infección por SARS-CoV-2, COVID de larga duración, síntomas clínicos y signos y síntomas; conforme figuran en los Descriptores en Ciencias de la Salud (DeCS) y en el *Medical Subject Headings* (MeSH) (Figura 1).

**Figura 1 -:** Estrategias de búsqueda utilizadas en las bases de datos. São Carlos, SP, Brasil, 2023

**Base de datos**	**Estrategia de búsqueda**
LILACS*	“Síndrome Pós-COVID-19^†^ Aguda” OR^‡^ “Afecções Pós-COVID” OR “COVID Longa” OR “Sequela Pós-Infecção por SARS-CoV-2^§^ Aguda” OR “Sequela Pós-Infecção por SARS-CoV-2 Aguda” OR “Post-Acute COVID-19 Syndrome” OR “Síndrome Post Agudo de COVID-19” OR “COVID de Longo Curso” AND^||^ “Sintomas Clínicos” OR “Sinais e Sintomas” OR “Sinais Clínicos” OR “Signs and Symptoms” OR “Signos y Síntomas”.
**PubMed** ^¶^	“Síndrome Pós-COVID-19 Aguda” OR “Afecções Pós-COVID” OR ” COVID Longa” OR “Sequela Pós-Infecção por SARS-CoV-2 Aguda” OR “Sequela Pós-Infecção por SARS-CoV-2 Aguda” OR “Post-Acute COVID-19 Syndrome” OR “Síndrome Post Agudo de COVID-19” OR “COVID de Longo Curso” NEAD/4^**^ “Sintomas Clínicos” OR “Sinais e Sintomas” OR “Sinais Clínicos” OR “Signs and Symptoms” OR “Signos y Síntomas”.
**Scopus**	“Síndrome Pós-COVID-19 Aguda” OR “Afecções Pós-COVID” OR ” COVID Longa” OR “Sequela Pós-Infecção por SARS-CoV-2 Aguda” OR “Sequela Pós-Infecção por SARS-CoV-2 Aguda” OR “Post-Acute COVID-19 Syndrome” OR “Síndrome Post Agudo de COVID-19” OR “COVID de Longo Curso” AND “Sintomas Clínicos” OR “Sinais e Sintomas” OR “Sinais Clínicos” OR “Signs and Symptoms” OR “Signos y Síntomas”.
Web of Science	“Síndrome Pós-COVID-19 Aguda” OR “Afecções Pós-COVID” OR “COVID Longa” OR “Sequela Pós-Infecção por SARS-CoV-2 Aguda” OR “Sequela Pós-Infecção por SARS-CoV-2 Aguda” OR “Post-Acute COVID-19 Syndrome” OR “Síndrome Post Agudo de COVID-19” OR “COVID de Longo Curso” AND “Sintomas Clínicos” OR “Sinais e Sintomas” OR “Sinais Clínicos” OR “Signs and Symptoms” OR “Signos y Síntomas”.

*LILACS = Literatura Latinoamericana y del Caribe en Ciencias de la Salud; ^†^Post-COVID-19 = Post *Corona Virus Disease 2019*; ^‡^OR = O; ^§^SARS-CoV-2 = *Severe Acute Respiratory Syndrome Coronavirus 2*; ^||^AND = Y; ^¶^PubMed = Public MedLars; ^**^NEAD/4 = Proximidad de hasta 4 términos

### Período

Las búsquedas de datos se realizaron entre los meses de mayo y junio de 2023.

### Criterios de selección

De esta forma, se aplicaron como criterios de inclusión: artículos originales, publicados en el período comprendido entre enero de 2020 y enero de 2023 y en los idiomas portugués, inglés y/o español, excluyéndose los artículos no disponibles en su totalidad, cuyos títulos y resúmenes no respondían a la cuestión orientadora, además de artículos de opinión, editoriales, revisiones y capítulos de libros. El período del estudio se refiere al inicio de la pandemia de COVID-19 y, consecuentemente, a las publicaciones relacionadas con la persistencia de los síntomas de esta enfermedad.

### Recolección de datos

Para llevar a cabo el presente estudio, se utilizó la estrategia PCC (Población, Concepto y Contexto), siendo “P” personas con COVID prolongada; “C” síntomas y “C” pandemia de COVID-19, definiéndose la siguiente cuestión orientadora: “¿Cuáles son los síntomas de COVID prolongada que presentan las personas, considerando las características clínicas y demográficas?”.

### Instrumentos utilizados para la recolección de información

Para la selección de los estudios, después de la implementación de la estrategia de búsqueda en las bases electrónicas mencionadas, los artículos fueron importados a la aplicación *web StArt* (*State of the Art through Systematic Review*), con el fin de realizar la selección de los estudios en dos niveles. Esta herramienta de revisión fue desarrollada por el Laboratorio de Investigación en Ingeniería de *Software* (LaPES) de la *Universidade Federal de São Carlos* (UFSCar)^(5)^. Así, durante la primera etapa de la selección, se realizó la lectura de títulos y resúmenes, seguida de la lectura completa de las referencias. Además, se verificaron las listas de referencias de todos los estudios encontrados.

### Tratamiento y análisis de los datos

Los estudios elegibles fueron recuperados en su totalidad y evaluados por tres investigadores, discutiendo las divergencias hasta llegar a un consenso para realizar la selección final. Se siguieron las directrices del PRISMA (*extension for scoping reviews*) para la extracción y presentación de los datos^(6)^. El proceso de selección de los estudios y la información relevante extraída de cada artículo seleccionado se presentan en figuras, en formato descriptivo.

### Aspectos éticos

Al tratarse de un estudio de revisión de alcance, no se requiere la aprobación por parte del Comité de Ética en Investigación.

## Resultados

Se importaron al *StArt* 536 artículos, de los cuales cuatro fueron excluidos por ser duplicados. Así, se consideraron 532 artículos para el análisis de título y resumen, de estos, 498 estudios fueron excluidos por no cumplir con los criterios de inclusión. De esta manera, se incluyeron para la lectura completa 34 artículos. Tras la lectura, 21 estudios fueron excluidos por no responder a la cuestión orientadora, siendo finalmente seleccionados 13 estudios (Figura 2).

En cuanto a los tipos de estudios incluidos en esta revisión, según la Figura 3, se destacan 8 (61,5%) estudios de cohorte, 3 (23,0%) estudios de caso, 1 (7,6%) caso-control y 1 (7,6%) estudio descriptivo. Además, en cuanto a los países de los estudios, 2 (15%) fueron realizados en los Estados Unidos; además, países como Arabia Saudita, Bélgica, Brasil, China, Egipto, España, Francia, Grecia, Italia, Londres y Suiza aparecen con solo 1 (7,6%) estudio cada uno.

**Figura 2 -:** Diagrama de flujo del proceso de selección de los artículos, según PRISMA-ScR. São Carlos, SP, Brasil, 2023

**Figura 3 -:** Descripción de los artículos según autor, año, lugar, objetivo, tipo de estudio, muestra y principales resultados. São Carlos, SP, Brasil, 2023

**Autor, año y lugar**	**Objetivo**	**Tipo de estudio y muestra (n)**	**Principales resultados**
Eita AAB, 2021, Estados Unidos de América^(7)^	Presentar un caso del síndrome post-agudo de COVID-19*, demostrando la parostomía, disgeusia y lengua grasa revestida de blanco con papilas prominentess	Estudio de caso. n = 1.	Después de 14 días de la infección por COVID-19*, una paciente femenina, de 31 años, con queja de disgeusia, presentaba lengua blanca grasa y desagradable, además de un olfato distorsionado. Los síntomas mejoraron a lo largo de cuatro semanas con tratamiento medicamentoso y tras la eliminación viral.
Judant EM, 2021, Francia^(8)^	Determinar las características de pacientes con quejas respiratorias y las relaciones entre disnea, anormalidades radiológicas y compromiso funcional	Estudio de cohorte. n = 478 (caso) y n =177 (control).	Individuos hospitalizados por COVID-19* fueron evaluados por consulta telefónica. La edad media de la muestra fue de 61 años y el 42,1% eran mujeres. Cuatro meses después del diagnóstico de la infección viral, 78 personas reportaron disnea y 23 reportaron tos. Comparativamente con los individuos sin disnea, aquellos con disnea eran más jóvenes (56,1±12,3 *versus* 61,9±16,6 años, p^†^=0,001). Los individuos con disnea presentaron episodios iniciales más graves de COVID-19*, con mayor tiempo de hospitalización [13 (7–23) *versus* 8 (4–14) días, p<0,001] e ingreso más frecuente en la Unidad de Cuidados Intensivos (56,4 *versus* 24,5%, p<0,001).
Moreno-Perez O, 2021, España^(9)^	Analizar la incidencia del síndrome post-agudo de COVID-19* y sus componentes y evaluar los factores de riesgo asociados a la fase aguda de la infección	Estudio de cohorte. n = 277.	Individuos con COVID-19* fueron evaluados 77 días después de la infección. La edad media fue de 62 años y el 52,7% eran hombres. Los síntomas post-COVID-19* fueron detectados en el 50,9% de los pacientes. Los síntomas más frecuentes fueron: disnea y fatiga. La anosmia-disgeusia se asoció a una edad más joven (<65 años 24,9% (48/194) vs >65 años 13,5% (11/83). Síntomas neurológicos relevantes (cefalea, trastornos de memoria/deterioro cognitivo o ambos) estuvieron presentes en 33 pacientes (11,9%). Tras ajuste multivariado, ninguna característica clínica basal, ingreso en la unidad de cuidados intensivos, tiempo de permanencia en el hospital o tratamiento se comportaron como predictores independientes del síndrome post-COVID-19*.
Penetra SLS, 2021, Brasil^(2022)^	Describir un caso de COVID-19* prolongado después de infección por SARS-CoV-2 Gamma un año después de la primera infección en un profesional de la salud vacunado con CoronaVac en Brasil	Estudio de caso. n = 1.	Paciente masculino, 43 años, sin comorbilidades. En la infección por COVID-19* presentó fiebre, cefalea, rinorrea y tos seca; los síntomas desaparecieron en 14 días. Nueve meses después de la infección, recibió la primera dosis de la vacuna y 28 días después la segunda dosis. Tras 67 días de la segunda dosis, desarrolló un nuevo episodio de COVID-19* de linaje Gamma. En esa ocasión, presentó los mismos síntomas; sin embargo, estos persistieron durante 18 semanas después de la infección aguda, excepto la cefalea y visión borrosa. En ambas infecciones no hubo necesidad de hospitalización.
Tleyjeh IM, 2021, Arabia Saudita^(11)^	Evaluar la frecuencia de síntomas nuevos o persistentes en pacientes con COVID-19* tras el alta hospitalaria e identificar factores de riesgo asociados	Estudio de cohorte. n = 222.	Se realizaron entrevistas telefónicas de seguimiento con individuos con COVID-19* después de una mediana de 122 días tras el alta hospitalaria. La mayoría de los pacientes eran hombres (77%) con una edad media de 52,47 (± 13,95) años. El tiempo de diagnóstico de los síntomas post-COVID-19* varió de 21 días a 4 meses tras el alta. El 56,3% de los pacientes se quejaron de persistencia de los síntomas; 66 (29,7%) los experimentaron durante >21 días y 64 (28,8%) reportaron no haber regresado a su salud inicial. Los síntomas más frecuentemente reportados fueron: falta de aire (40,1%), tos (27,5%) y fatiga (29,7%). Tras ajustes multivariados, el sexo femenino, la hipertensión preexistente y el tiempo de hospitalización se asociaron a un mayor riesgo de síntomas nuevos o persistentes. La edad, la enfermedad pulmonar preexistente y las visitas a los servicios de urgencia aumentaron la probabilidad de no recuperación completa del COVID-19* agudo.
Tohamy D, 2021, Egipto^(12)^	Evaluar las manifestaciones oculares del síndrome post-agudo de COVID-19*	Estudio de cohorte. n = 100 (caso) y n = 100 (control).	El tiempo de diagnóstico de las condiciones post-COVID-19* varió de 1 a 3 meses. En la muestra, los individuos no tenían antecedentes de vacunación contra COVID-19*. La media de edad fue de 55,5 años en el grupo COVID frente a 56,5 años en el grupo control. En el grupo COVID, el 57% de los pacientes eran hombres frente al 51% en el grupo control. En el grupo COVID, 5 pacientes presentaban oclusión vascular retiniana, 2 pacientes presentaban neuropatía óptica isquémica anterior, 3 pacientes presentaban uveítis y 2 pacientes presentaban coriorretinopatía serosa central. Mientras que en el grupo control, 2 pacientes presentaban oclusión vascular retiniana y ninguno presentaba uveítis o coriorretinopatía serosa central.
Carter SJ, 2022, Estados Unidos de América^(13)^	Describir los efectos concomitantes de la infección por SARS-CoV-2 en cuanto al estado funcional, estado de ánimo y actividad física de ocio en el síndrome post-agudo de COVID-19*	Estudio caso-control. n =17 (caso) y n =15 (control).	El tiempo de diagnóstico de las condiciones post-COVID-19* fue de aproximadamente 4 semanas. Se incluyeron 32 mujeres, 17 diagnosticadas con COVID-19* y 15 controles. La presión arterial diastólica (*p* = 0,031, ES = 0,80) y la RPP^‡^ (*p* = 0,039, ES = 0,75) fueron significativamente mayores entre los participantes del grupo COVID-19*. En el grupo COVID-19*, se observaron los siguientes síntomas: 41% con dolores articulares/musculares por cerca de 300 días, 53% con tos en el mismo periodo; 29% con fatiga, 12% con deterioro cognitivo y 71% con pérdida de gusto/olfato en un periodo aproximado de 100 días, además de 35% con dificultad para respirar.
Fanous J, 2022, Londres^(14)^	Evaluar objetivamente la fatigabilidad neuromuscular concomitante a fatiga y disfunción cognitiva relacionada con COVID-19* tras 12 meses de la infección	Estudio de caso. n = 1.	El tiempo de diagnóstico de las condiciones post-COVID-19* fue de cerca de 12 meses. Paciente masculino, 27 años, con queja continua post-COVID-19* de fatiga y disfunción cognitiva, anosmia y ageusia, fuerte confusión mental, pérdida de apetito e insomnio más de un año después de la infección.
Jamoulle M, 2022, Bélgica^(1)^	Describir de manera cualitativa y cuantitativa el estado de salud de los pacientes con Síndrome Post-Agudo de COVID-19*	Estudio de cohorte. n = 55.	El tiempo de diagnóstico de las condiciones post-COVID-19* varió de 3 a 27 meses. El 12% de la muestra no fue vacunada durante el periodo del estudio. Entre los pacientes, el 72,7% eran mujeres, con edades comprendidas entre 12 y 79 años. Después de un año de seguimiento, se pudieron distinguir tres tipos de evolución clínica desde el inicio de la COVID-19* aguda en 52 de los 55 pacientes: Grado 1 (leve) - duración de 3 a 8 meses, compromiso respiratorio, fatiga, dolor esternal y agotamiento con esfuerzo (9 mujeres y 7 hombres); Grado 2 (grave) - duración de 6 a 18 meses, fatiga extrema, agotamiento al esfuerzo, anosmia, trastorno cognitivo, trastorno de memoria (12 mujeres y 3 hombres); Grado 3 (muy grave) - duración de 12 a 27 meses, agotamiento constante, esfuerzos imposibles, revalidación cognitiva ineficaz, hipersomnia, aumento de peso por inactividad, graves y persistentes trastornos de memoria y considerable ansiedad. Otros síntomas variables mencionados incluyeron niebla mental con compromiso de la memoria y déficit en la recuperación de palabras, disnea al esfuerzo y estado incapacitante.
Kim Y, 2022, Corea del Sur^(15)^	Identificar síntomas prevalentes después de 12 meses de la infección por COVID-19* en pacientes recuperados, factores asociados a los síntomas neurológicos e impacto a largo plazo de COVID-19 en la calidad de vida	Estudio de cohorte. n = 900 (caso) y n = 241 (control)	El tiempo de diagnóstico de las condiciones post-COVID-19* varió de 6 a 12 meses. La edad media de los respondientes fue de 37 años y 164 eran mujeres. Los síntomas persistentes por más de 12 meses fueron mayores en aquellos clasificados con gravedad moderada o mayor de la enfermedad durante la infección aguda por COVID-19*. El síntoma más frecuente que duró hasta 12 meses fue dificultad de concentración, observado en 54 personas, seguido de disfunción cognitiva (51), amnesia (48), depresión (43) y fatiga y ansiedad (39). Entre los síntomas persistentes relacionados con COVID-19*, los problemas psiquiátricos tuvieron un mayor impacto en la calidad de vida que cualquier otro síntoma. Edad avanzada, sexo femenino y gravedad de la enfermedad fueron identificados como factores de riesgo para síntomas neuropsiquiátricos persistentes.
Liu Q, 2022, China^(16)^	Investigar si la composición de la microbiota intestinal está vinculada al síndrome post-agudo	Estudio de cohorte. n = 106 (caso) y n = 68 (control)	El tiempo de diagnóstico de las condiciones post-COVID-19* varió de 3 a 6 meses. Los pacientes con agravamiento de COVID-19* fueron seguidos desde la admisión hasta seis meses. La edad media del grupo COVID fue de 48,3 años y el 52,9% eran mujeres. Se verificaron las siguientes comorbilidades: 17% hipertensión y 15,1% diabetes mellitus. El 81,1% de los pacientes reportaron síndrome post-agudo de COVID-19* en 3 meses. Los síntomas más comunes fueron: fatiga (31,3%), memoria débil (28,3%), caída del cabello (21,7%), ansiedad (20,8%) y dificultad para dormir (20,8%). No hubo diferencias significativas en edad, sexo, comorbilidades, uso de medicamentos ni gravedad de COVID-19* en pacientes con o sin síndrome post-agudo de COVID-19*.
Mazzitelli M, 2022, Italia^(17)^	Evaluar la prevalencia y los factores asociados a COVID-19* grave y síndrome post-agudo de COVID-19*	Estudio de cohorte. n = 123.	El tiempo de diagnóstico de las condiciones post-COVID-19* fue de 6 meses. Se analizaron pacientes diagnosticados con VIH^§^ y, concomitantemente, con COVID-19*. De ellos, el 79,7% eran hombres, el 77,2% de etnia caucásica y la edad media fue de 51 años. Ninguno de los pacientes había sido vacunado contra COVID-19*. En cuanto a comorbilidades: el 30,9% eran hipertensos, el 17,9% diabéticos y el 15,4% tenían sobrepeso/obesidad; de los 75 pacientes que presentaron infección sintomática de COVID-19* y que sobrevivieron, el 26,7% reportó síndrome post-agudo de COVID-19*. En cuanto a los síntomas: el 80% reportó astenia; el 50% falta de aire al esfuerzo; el 25% dolor de cabeza recurrente; el 15% caída significativa del cabello; el 10% pérdida del olfato; el 0,5% diarrea crónica; además, 2 pacientes reportaron complicaciones cardiovasculares (infarto agudo de miocardio y alteraciones rítmicas).
Zachariou A, 2022, Grecia^(18)^	Evaluar las consecuencias en la función de la vejiga en pacientes con síndrome post-agudo de COVID-19* transferidos a la rehabilitación de pacientes internados para cuidados prolongados tras el tratamiento inicial de la fisiopatología de COVID-19*	Estudio descriptivo. n = 147.	El tiempo de diagnóstico de las condiciones post-COVID-19* varió de 4 a 7 semanas. Estudio realizado con pacientes con síndrome post-agudo de COVID-19* derivados al centro de rehabilitación para cuidados prolongados. De estos, solo 106 aceptaron participar en la investigación, siendo 45 (43,4%) mujeres y 60 (56,6%) hombres. La edad media de los pacientes fue de 59,5 años. Se identificaron 75 pacientes con síntomas asociados a la vejiga hiperactiva (VH) entre 4 a 7 semanas tras COVID-19*. De estos, 9 pacientes se negaron a responder los cuestionarios; 44 pacientes tuvieron VH recién diagnosticada y 22 pacientes presentaron un empeoramiento de los síntomas de VH. Todos los pacientes reportaron nocturia.

*COVID-19 = *Corona Virus Disease 2019;* ^†^p = Nivel de significancia; ^‡^RPP = *Rate-Pressure Product*; ^§^HIV = *Human Immuno-Deficiency Virus*

En cuanto a los datos demográficos, se verificó que en 5(38,4%) estudios la media de edad de los casos de COVID Prolongado varió entre 42,4 y 55,5 años. En los estudios con muestras compuestas por ambos sexos, cuatro describieron sus muestras con los mayores porcentajes correspondientes a hombres y en tres estudios predominó el sexo femenino.

Entre los síntomas más frecuentemente observados, se destacaron la fatiga (61,5%)^(1,7,9,11,13-16)^, disnea (46,1%)^(1,8-9,11,13,17)^, alteraciones en el olfato y/o gusto (38,6%)^(1,7,9,13,17)^, ansiedad (15,3%)^(1,15)^ y deterioro cognitivo (30,7%)^(1,13-14,16)^.

En relación con las características clínicas de las personas con síntomas sugestivos de COVID Prolongado y la presencia de enfermedades preexistentes, solo 5 (38,4%) estudios describieron las comorbilidades más frecuentes observadas en sus muestras, siendo predominante el reporte de diabetes mellitus e hipertensión^(9,11,15-17)^.

Una variable clínica poco abordada en los estudios fue la relación entre el esquema de vacunación contra COVID-19 y el desarrollo de COVID Prolongado. Se verificó que 9 (69,24%) estudios no mencionaron la situación de vacunación^(7-9,11,13-16,18)^, 2 (15,3%) afirmaron que la población del estudio no estaba vacunada^(12,17)^ y 1 (7,6%) mostró que aproximadamente el 12% de la población investigada no había sido vacunada durante el periodo del estudio^(1)^. Ninguno de los estudios analizó la asociación entre la vacunación y el desarrollo de síntomas de COVID Prolongado.

Otras lagunas identificadas en los estudios seleccionados se refieren a la ausencia de análisis de la asociación de COVID Prolongado con variables demográficas, como escolaridad e ingresos^(1,7-18)^, o variables clínicas sobre antecedentes de hospitalización por COVID-19^(1,7,12-14,18)^.

En cuanto a los factores de riesgo asociados al desarrollo de COVID Prolongado, solo dos estudios describieron una asociación positiva relacionada con la gravedad de la fase aguda de la enfermedad COVID-19, la presencia de comorbilidades, mayor tiempo de hospitalización e ingreso en Unidad de Cuidados Intensivos, edad avanzada y sexo femenino con el mayor riesgo de presentar algunos síntomas de COVID Prolongado^(8,11,15)^. Por otro lado, un estudio indicó que la presencia de anosmia-disgeusia se asoció a edades más jóvenes (menores de 65 años)^(9)^.

## Discusión

A partir del análisis de los estudios seleccionados en esta revisión, se identificó una amplia gama de síntomas reportados en personas que desarrollaron la enfermedad COVID Prolongado. Entre los más frecuentes se destacaron la fatiga, disnea, alteraciones en el olfato y gusto, y trastornos cognitivos relacionados con la memoria. Además, se señaló que enfermedades como hipertensión arterial y diabetes mellitus fueron comorbilidades comúnmente encontradas en las personas. En este análisis, se observó una mayor prevalencia de COVID Prolongado en muestras de personas adultas de ambos sexos. Sin embargo, algunos estudios asociaron el sexo femenino como un factor de riesgo para el desarrollo de la enfermedad, y otros factores relacionados con un mayor riesgo de desarrollar COVID Prolongado fueron la gravedad de la infección por COVID-19, mayor tiempo de hospitalización y edad avanzada. Asimismo, en relación con el esquema de vacunación contra la COVID-19, se observó la ausencia de esta temática en la mayoría de los estudios analizados sobre la manifestación de síntomas en COVID Prolongado.

Cabe destacar que en el primer año de la pandemia, en 2020, el término *Long COVID* se utilizó para describir a los pacientes que se recuperaron de la enfermedad, pero que aún presentaban efectos duraderos de la infección por un tiempo mayor al esperado^(19)^. Además, el grado de infección por COVID-19 podría estar relacionado con los diferentes síntomas manifestados en el desarrollo de COVID Prolongado. De este modo, COVID Prolongado se observó con mayor frecuencia en personas que desarrollaron una forma grave de COVID-19 en comparación con casos menos graves^(20)^. Aún, el riesgo de desarrollar COVID Prolongado aumenta con la edad, pero puede afectar a toda la población, incluyendo al público infantil^(21-22)^.

Aunque la fisiopatología de COVID Prolongado no está completamente aclarada, entre las hipótesis explicativas de la enfermedad se mencionan la presencia de reservorios persistentes de SARS-CoV-2 en tejidos, desregulación inmunológica, impactos del virus en la microbiota, autoinmunidad, preparación del sistema inmunológico a partir del mimetismo molecular, coagulación sanguínea microvascular con disfunción endotelial y señalización disfuncional en el tronco encefálico y/o nervio vago^(22-23)^.

En cuanto a los síntomas manifestados en COVID Prolongado, se ha descrito que estos pueden superponerse a diversas condiciones no relacionadas directamente con la infección por COVID-19, como por ejemplo, el síndrome post cuidados intensivos en casos que requirieron hospitalización, o incluso la exacerbación de condiciones de salud preexistentes^(24-25)^.

En este contexto, los síntomas manifestados en COVID Prolongado pueden tener etiologías distintas. Así, en el abordaje de los síntomas respiratorios a largo plazo, estos podrían ser causados por trastornos vasculares pulmonares resultantes del daño vascular en los microvasos pulmonares sufrido durante la infección aguda por SARS-CoV-2. Uno de los síntomas más reportados, la disnea, en ausencia de lesión pulmonar, podría estar relacionado con una regulación inadecuada de la ventilación resultante de trastornos del sistema nervioso autónomo, con posibles daños en los receptores reflejos intratorácicos o en el tronco encefálico/zonas corticales cerebrales^(23,26)^. Por otro lado, las manifestaciones de dolor, incluyendo dolor articular y muscular, podrían atribuirse a mecanismos tromboinflamatorios en relación con lesiones tisulares y procesos autoinmunes^(27-28)^.

En cuanto a los síntomas neurológicos presentados en COVID Prolongado, un análisis señaló que las secuelas persistentes podrían estar relacionadas con complicaciones neurológicas agudas en la infección por SARS-CoV-2, resultantes de daño cerebral u otros factores relacionados con la hospitalización. De este modo, la manifestación de trastornos cognitivos, cefalea y alteraciones olfativas y/o gustativas, podría depender de una fisiopatología independiente de la fase aguda. Una explicación sería que la propagación del virus SARS-CoV-2 en el cerebro, por medio de la cavidad nasal o del torrente sanguíneo, resultaría en una neuroinflamación, y esta, siendo persistente, podría ser responsable del deterioro neurocognitivo o de trastornos de salud mental^(23)^.

De esta manera, los exámenes clínicos de personas con COVID Prolongado muestran una disminución de serotonina causada por la infección de COVID-19 debido a la reducción de la absorción de triptófano, trombocitopenia y aumento en la expresión de la flavoenzima monoaminoxidasa, lo que resulta en una disminución de la activación vagal e hipocampal y, también, en un considerable deterioro cognitivo. Además, se constató que la reducción de serotonina y la disfunción del nervio vago pueden estar asociadas a COVID Prolongado, ya que sugieren un curso posterior a la infección viral^(29)^.

No obstante, a partir de la idea de que la fenotipificación metabolómica, proteómica e inmunológica de pacientes que contrajeron SARS-CoV-2 se asoció a una diversidad de síntomas clínicos con potenciales biomarcadores para COVID-19, el metabolismo desregulado y la inflamación podrían contribuir a los síntomas de COVID Prolongado. En este contexto, se destaca que los triglicéridos sanguíneos totales y los metabolitos lactato y piruvato fueron más elevados y las lipoproteínas más bajas en pacientes con COVID Prolongado en comparación con los controles sanos^(30)^.

Asimismo, se pudo verificar que las citocinas o el sexo femenino y/o masculino están relacionados con los metabolitos, como citrato, glutamato e histidina, en pacientes que presentaron síntomas como fatiga crónica, disnea y confusión mental en el curso prolongado de la enfermedad. En fin, varias citocinas y quimiocinas se correlacionaron con metabolitos y lipoproteínas, considerándose la desregulación en el metabolismo y la inflamación como factores potenciales para el desarrollo de COVID Prolongado^(30)^.

Entre los factores de riesgo para el desarrollo de COVID Prolongado, se describieron la hospitalización debido a la infección, el aumento de la edad, el sexo femenino y la presencia de comorbilidades^(31)^. En esta perspectiva, se sabe que enfermedades comúnmente encontradas en la población, como la diabetes, cuando no está bien controlada, causan daños en los órganos, ocasionando específicamente lesiones microvasculares que pueden verse exacerbadas en la infección por SARS-CoV-2 en su forma grave. Además, el estado inflamatorio preexistente puede mantenerse exacerbado y agravarse después de la infección^(32)^.

También, los síndromes coronarios agudos, insuficiencia cardíaca, arritmias, accidente cerebrovascular y tromboembolismo persisten más allá de la etapa inicial de la infección por SARS-CoV-2, prolongándose durante varios meses. Síntomas cardiopulmonares como dolor en el pecho, dificultad para respirar, fatiga y taquicardia ortostática postural pueden ser comunes en COVID Prolongado y están asociados a una discapacidad significativa y un aumento de la ansiedad. Sin embargo, los mecanismos fisiopatológicos para las complicaciones cardiovasculares tardías no están bien comprendidos^(33)^.

La literatura señala divergencias en cuanto a la prevalencia de COVID Prolongado. Un estudio que analizó la población del norte de los Países Bajos describió que la enfermedad puede ocurrir en alrededor de 1 de cada 8 personas con la enfermedad en la población general, mientras que otra investigación en Italia informó que 9 de cada 10 pacientes, tras recuperarse de la enfermedad, aún presentaban al menos un síntoma después de 60 días desde el inicio^(19,34)^.

Además, la prevalencia de COVID Prolongado difiere entre hombres y mujeres, considerando las diferencias fisiológicas y hormonales que influyen en el sistema inmunológico. Así, las mujeres tienen niveles más altos de inmunoglobulina, lo que hace que desarrollen respuestas inmunológicas más fuertes tras la inmunización o infección en comparación con los hombres, aunque presentan una mayor susceptibilidad a las enfermedades autoinmunes^(35)^.

De este modo, las mujeres pueden presentar diferentes factores de riesgo para COVID Prolongado dependiendo de su estado hormonal. En este contexto, la menopausia es un fenómeno que se asocia a un mayor riesgo de la enfermedad, ya que las mujeres en posmenopausia presentan una mayor incidencia de COVID-19 en comparación con las mujeres en premenopausia. Así, se sugiere que el estrógeno podría estar asociado con la gravedad de la enfermedad; además, se destaca que la ECA2 se encuentra en el cromosoma X y que el estrógeno es un regulador negativo de la expresión de ECA2. También, el receptor Toll-like 7 (TLR7), regulador de la producción de interferón (IFN), es considerado un gen inmunitario del cromosoma X que provoca una mayor señalización de IFN en COVID-19 y una mejor depuración viral en mujeres; sin embargo, la señalización continua las predispone a un mayor riesgo de desarrollar COVID Prolongado^(36)^.

No obstante, en ambos sexos, los hallazgos clínicos, las alteraciones en las células T, la reducción en el número de células de memoria efectora *cluster of differentiation* 4+ (CD4+) y *cluster of differentiation* 8+ (CD8+) y la alta expresión de la proteína PD1 en células de memoria central, han persistido durante al menos 13 meses en individuos con COVID Prolongado que tuvieron COVID-19 leve^(22,37)^.

En individuos que desarrollaron COVID Prolongado se detectó una disminución de inmunoglobulina M (IgM) desde la infección primaria hasta el seguimiento a 6 meses. Además, mientras que la inmunoglobulina G1 (IgG1) permaneció inalterada, la inmunoglobulina G3 (IgG3) fue menor en pacientes con COVID Prolongado, ocurriendo lo contrario en las concentraciones de IgG3 en casos leves y graves de COVID-19. Así, fue posible observar una firma de inmunoglobulina que, combinada con la edad, el historial de asma bronquial y cinco síntomas durante la infección primaria, puede predecir el riesgo de COVID Prolongado independientemente del periodo en que se haya realizado la recolección de sangre^(38)^.

Por medio de títulos de inmunoglobulina G (IgG) específicos para SARS-CoV-2 distribuidos equitativamente en un periodo según edad, sexo y presencia/ausencia de síntomas prolongados, los pacientes fueron vacunados entre el décimo y decimoquinto mes tras la infección, identificando que la administración temprana de la vacuna es más eficaz que la tardía. Además, la vacunación administrada antes de las infecciones por SARS-CoV-2 está asociada con una menor prevalencia de síntomas persistentes^(39)^.

En este contexto, aunque la vacunación contra COVID-19 no fue una variable clínica analizada en los estudios seleccionados en esta revisión, su relación con COVID Prolongado merece destaque. La literatura señala que la vacunación doble produjo un efecto beneficioso temporal en los síntomas prolongados, con una duración de 21 a 67 días^(39)^. En una revisión sistemática que analizó datos sobre la vacunación antes y después de la infección por SARS-CoV-2, en relación con la vacunación antes de la infección, se mostró que los individuos presentaron una reducción significativa en la incidencia de COVID Prolongado. Por otro lado, al analizar la remisión y recuperación de COVID Prolongado, existen posibilidades de no recuperación cuando los pacientes fueron vacunados después de la infección^(40)^.

Así, la vacunación contra COVID-19, además de actuar en la reducción de la gravedad de la enfermedad y las tasas de hospitalización, ha sido asociada a una considerable disminución en el desarrollo de síntomas de COVID Prolongado^(40-41)^. En esta perspectiva, es notoria la importancia de adherirse a la vacunación contra COVID-19 ya que, al ser reconocida, actúa como un instrumento de protección contra el desarrollo de la enfermedad prolongada, considerando que aún no existen tratamientos ampliamente eficaces tanto para la enfermedad aguda como para COVID Prolongado^(22)^.

Finalmente, es importante destacar que la atención sanitaria destinada a las personas con COVID Prolongado puede estar comprometida, especialmente en países de ingresos bajos y medios, debido a sistemas de salud limitados y con pocos recursos, lo que podría representar una gran demanda para los sistemas de salud ya sobrecargados y agravar aún más las desigualdades en salud^(42)^.

Esta revisión de alcance contribuye a la identificación de los síntomas más frecuentes manifestados en COVID Prolongado, entre ellos fatiga, disnea, alteraciones en el olfato y gusto, y trastornos cognitivos relacionados con la memoria. Además, se observó en los estudios seleccionados una mayor prevalencia de COVID Prolongado en personas correspondientes al grupo de edad adulta y de ambos sexos.

Entre las limitaciones del estudio se encuentran la inclusión de solo estudios en portugués, inglés y español, artículos disponibles en texto completo y bases de indexación no incluidas en esta investigación.

## Conclusión

El mapeo de la literatura evidenció la diversidad de síntomas en personas que desarrollaron COVID Prolongado, siendo más prevalentes en personas adultas, de ambos sexos y con presencia de comorbilidades. Debido a la elevada carga de síntomas persistentes ocasionados tras la infección por SARS-CoV-2, COVID Prolongado representa un desafío para los servicios de salud, especialmente en cuanto a la diversidad de manifestaciones sintomáticas. Esta situación implica dificultades para la calidad de vida de las personas afectadas por COVID Prolongado.

Esta revisión encontró importantes lagunas que pueden influir en la comprensión de COVID Prolongado; de este modo, se recomienda que futuros estudios aborden la gravedad y persistencia de los síntomas presentados en COVID Prolongado y la relación de la adherencia al esquema de vacunación contra COVID-19, sexo, raza/etnia, grado de susceptibilidad en diferentes grupos etarios, nivel educativo e ingresos, además de las comorbilidades más recurrentes en la población. La profundización de estudios utilizando estas variables puede contribuir a la efectividad de estrategias de salud pública para el diagnóstico y la asistencia a personas afectadas por esta enfermedad.

# Translation: Sintomas prevalentes e características da população na COVID Longa: uma revisão de escopo*

**DOI:** 10.1590/1518-8345.7353.4480

**Authors:** Karina Marques Prediger, Ana Cristina Ribeiro, Sílvia Carla da Silva André Uehara

**Affiliations:** 1 Universidade Federal de São Carlos, Departamento de Enfermagem, São Carlos, SP, Brasil; 2 Bolsista da Fundação de Amparo à Pesquisa do Estado de São Paulo (FAPESP), Brasil

**Keywords:** Síndrome de COVID-19 Pós-Aguda, COVID-19, Sinais e Sintomas, Características da População, Pandemias, Revisão

## Abstract

mapear a literatura científica sobre as características clínicas e demográficas da COVID Longa.

trata-se de uma revisão de escopo baseada nos princípios preconizados pelo JBI e as diretrizes do PRISMA para a extração dos dados, realizada a partir de quatro bases de dados. Utilizou-se a estratégia PCC para a coleta de dados, sendo os resultados descritos e diagramados. A seleção dos estudos foi realizada após a remoção de duplicatas, avaliação individual e em pares.

uma análise dos 13 artigos selecionados mostraram que a COVID Longa atinge todas as faixas etárias e pessoas de ambos os sexos, apresentando uma multiplicidade de sintomas, como fadiga (61,5%), dispneia (46,1%), alterações no olfato e/ou paladar (38,6%), ansiedade (15,3%) e comprometimento cognitivo (30,7%). No sexo feminino foi identificado risco aumentado para desenvolver COVID Longa.

a identificação dos sintomas prevalentes na COVID Longa contribuem na estratégia de saúde pública, para o diagnóstico e assistência às pessoas acometidas pela doença. Recomenda-se estudos futuros sobre a abordagem da persistência dos sintomas na COVID Longa e a relação da adesão ao esquema vacinal contra a COVID-19, sexo, raça/etnia, grau de susceptibilidade nas diferentes faixas etárias, nível de escolaridade e renda além das comorbidades mais recorrentes na população.

## Destaques

**(1)** Identificou-se que a COVID Longa acomete todas as faixas etárias de ambos os sexos.

**(2)** Sintomas mais frequentes: fadiga, dispneia e alteração no olfato e/ou paladar.

**(3)** Fatores de risco: sexo feminino, gravidade de COVID-19 e comorbidades.

## Introdução

A doença do novo coronavírus (COVID-19) não se restringe apenas a uma doença aguda, sendo que aproximadamente 10 a 50% dos pacientes infectados podem evoluir para a COVID Longa, sendo caracterizada por uma doença multissistêmica com sintomas heterogêneos^(1)^. A COVID Longa pode ocorrer em indivíduos com infecção provável ou confirmada ocasionada pelo vírus denominado *Severe Acute Respiratory Syndrome Coronavirus 2* (SARS-CoV-2)^(2)^.

A COVID Longa comumente é observada após três meses do diagnóstico da infecção na fase aguda e os sintomas persistem por pelo menos dois meses e não podem ser explicados por um diagnóstico alternativo. Além disso, os sintomas podem reaparecer após a recuperação inicial de um episódio agudo de COVID-19 ou persistir desde a doença inicial; ainda, os sintomas podem flutuar ou recidivar entre o período^(2)^.

Nesse cenário, a COVID-19 pode afetar vários sistemas orgânicos e ocasionar uma série de sintomas que não se restringem apenas à região pulmonar. Desse modo, há um número significativo de pessoas que apresentam sintomas que vão desde fadiga grave até distúrbios neurológicos, e vivenciam as consequências negativas desse fenômeno, ao passo que sofrem prejuízos em sua qualidade de vida^(3)^.

Concomitantemente, a COVID Longa apresenta uma maior prevalência entre pacientes comórbidos que relatam fadiga, nevoeiro cerebral e mialgia enquanto principais sintomas, podendo ser múltiplos e de variável intensidade, a depender da área do corpo afetada. Ainda, o profissional de saúde, ao atender um paciente que apresenta tais sintomas, pode associá-los às causas psicossomáticas como depressão e ansiedade, conduta essa que dificulta o diagnóstico de COVID Longa^(1)^.

Sendo assim, os mecanismos fisiopatológicos da COVID Longa ainda não estão totalmente esclarecidos, a diversidade e complexidade dos sintomas dificultam sua categorização, o que contribui para a lacuna do conhecimento quanto ao diagnóstico e ao tratamento da doença. Portanto, são essenciais estudos que abordem essa temática para contribuir na implementação de estratégias de saúde pública e auxiliar os serviços de saúde na identificação dos principais sintomas manifestados e quais pessoas se apresentam mais suscetíveis ao desenvolvimento da COVID Longa.

Portanto, tratar a doença para além de seu curso agudo requer investigações que visem identificar a sintomatologia e a fisiopatologia da COVID Longa, bem como formas de assegurar um diagnóstico preciso com vistas a oferecer uma assistência qualificada e efetiva. Dessa forma, este estudo visa mapear a literatura científica sobre as características clínicas e demográficas na COVID Longa.

## Método

### Tipo do estudo

Foi realizada uma *Scoping Review* (SR) com base nos princípios e fases preconizadas pelo *Joanna Briggs Institute* (JBI), a saber: (1) identificação da questão de pesquisa; (2) identificação de estudos relevantes; (3) seleção dos estudos; (4) extração de dados; (5) separação, sumarização e relatório de resultados; e (6) divulgação dos resultados^(4)^. O protocolo deste estudo está publicado na *Open Science Framework*: https://doi.org/10.17605/OSF.IO/QRS4J.

### Cenário

A pesquisa bibliográfica foi conduzida nas bases de dados eletrônicas: LILACS, PubMed, Scopus e *Web of Science*. As buscas foram conduzidas pelos descritores e/ou termos alternativos: síndrome pós-COVID-19 aguda, sequela pós-infecção por SARS-CoV-2, COVID de longo curso, sintomas clínicos e sinais e sintomas; conforme consta no Descritores em Ciências da Saúde (DeCS) e no *Medical Subject Headings (*MeSH) (Figura 1).

**Figura 1 -:** Estratégias de busca utilizadas nas bases de dados. São Carlos, SP, Brasil, 2023

**Base de dados**	**Estratégia de busca**
LILACS*	“Síndrome Pós-COVID-19^†^ Aguda” OR^‡^ “Afecções Pós-COVID” OR “COVID Longa” OR “Sequela Pós-Infecção por SARS-CoV-2^§^ Aguda” OR “Sequela Pós-Infecção por SARS-CoV-2 Aguda” OR “Post-Acute COVID-19 Syndrome” OR “Síndrome Post Agudo de COVID-19” OR “COVID de Longo Curso” AND^||^ “Sintomas Clínicos” OR “Sinais e Sintomas” OR “Sinais Clínicos” OR “Signs and Symptoms” OR “Signos y Síntomas”.
**PubMed** ^¶^	“Síndrome Pós-COVID-19 Aguda” OR “Afecções Pós-COVID” OR ” COVID Longa” OR “Sequela Pós-Infecção por SARS-CoV-2 Aguda” OR “Sequela Pós-Infecção por SARS-CoV-2 Aguda” OR “Post-Acute COVID-19 Syndrome” OR “Síndrome Post Agudo de COVID-19” OR “COVID de Longo Curso” NEAD/4^**^ “Sintomas Clínicos” OR “Sinais e Sintomas” OR “Sinais Clínicos” OR “Signs and Symptoms” OR “Signos y Síntomas”.
**Scopus**	“Síndrome Pós-COVID-19 Aguda” OR “Afecções Pós-COVID” OR ” COVID Longa” OR “Sequela Pós-Infecção por SARS-CoV-2 Aguda” OR “Sequela Pós-Infecção por SARS-CoV-2 Aguda” OR “Post-Acute COVID-19 Syndrome” OR “Síndrome Post Agudo de COVID-19” OR “COVID de Longo Curso” AND “Sintomas Clínicos” OR “Sinais e Sintomas” OR “Sinais Clínicos” OR “Signs and Symptoms” OR “Signos y Síntomas”.
Web of Science	“Síndrome Pós-COVID-19 Aguda” OR “Afecções Pós-COVID” OR “COVID Longa” OR “Sequela Pós-Infecção por SARS-CoV-2 Aguda” OR “Sequela Pós-Infecção por SARS-CoV-2 Aguda” OR “Post-Acute COVID-19 Syndrome” OR “Síndrome Post Agudo de COVID-19” OR “COVID de Longo Curso” AND “Sintomas Clínicos” OR “Sinais e Sintomas” OR “Sinais Clínicos” OR “Signs and Symptoms” OR “Signos y Síntomas”.

*LILACS = Literatura Latino-Americana e do Caribe em Ciências da Saúde; ^†^Pós-COVID-19 = Pós *Corona Virus Disease 2019*; ^‡^OR = Ou; ^§^SARS-CoV-2 =*Severe Acute Respiratory Syndrome Coronavirus 2*; ^||^AND = E; ^¶^PubMed = Public MedLars; ^**^NEAD/4 = Proximidade em até 4 termos

### Período

As buscas dos dados ocorreram entre os meses de maio a junho de 2023.

### Critérios de seleção

Os critérios de inclusão aplicados foram: artigos originais, publicados no período compreendido de janeiro de 2020 a janeiro de 2023 e nos idiomas português, inglês e/ou espanhol. Foram excluídos artigos não disponíveis na íntegra, cujo títulos e resumos não respondiam à questão norteadora, além de artigos de opiniões, editoriais, revisões e capítulos de livros. O período do estudo se refere ao início da pandemia por COVID-19 e, consequentemente, de publicações relacionadas à persistência dos sintomas dessa doença.

### Coleta de dados

A fim de conduzir o presente estudo, utilizou-se a estratégia PCC (População, Conceito e Contexto), sendo “P” pessoas com COVID Longa; “C” sintomas e “C” pandemia de COVID-19, sendo definida a seguinte questão norteadora: “Quais os sintomas de COVID Longa são apresentados pelas pessoas, considerando as características clínicas e demográficas?”.

### Instrumentos utilizados para a coleta de dados das informações

Para a seleção dos estudos, após implementação da estratégia de busca nas bases eletrônicas citadas, os artigos foram importados para o aplicativo *web StArt (State of the Art through Systematic Review),* a fim de se realizar a seleção dos estudos em dois níveis. Essa ferramenta de revisão foi desenvolvida pelo Laboratório de Pesquisa em Engenharia de *Software* (LaPES) da Universidade Federal de São Carlos (UFSCar)^(5)^. Assim, durante a primeira etapa da seleção, realizou-se a leitura de títulos e resumos, seguida da leitura na íntegra das referências. Ainda, foram verificadas as listas de referências de todos os estudos encontrados.

### Tratamento e análise dos dados

Os estudos elegíveis foram recuperados na íntegra e avaliados por três pesquisadores, sendo que as divergências foram discutidas até se chegar a um consenso e, então, realizar-se a seleção final. Foram seguidas as diretrizes do PRISMA (*extension for scoping reviews*) para a extração e apresentação dos dados^(6)^. O processo de seleção dos estudos e as informações relevantes extraídas de cada artigo selecionado estão apresentados em figuras, no formato descritivo.

### Aspectos éticos

Por se tratar de um estudo de revisão de escopo, é dispensado a aprovação pelo Comitê de Ética em Pesquisa.

## Resultados

Foram importados para o *StArt* 536 artigos, dos quais quatro foram excluídos por serem duplicados. Assim, considerou-se 532 artigos para análise de título e resumo, destes, 498 estudos foram excluídos por não se enquadrar nos critérios de inclusão. Dessa forma, 34 artigos foram incluídos para a leitura na íntegra. Após a leitura, excluíram-se 21 estudos por não responderem à questão norteadora, sendo selecionados, ao final 13, estudos (Figura 1).

Em relação aos tipos de estudos incluídos nesta revisão, conforme a Figura 3, destacam-se 8 (61,5%) estudos de coorte, 3 (23,0%) estudos de caso, 1 (7,6%) caso-controle e 1 (7,6%) estudo descritivo. Ainda, quanto aos países de estudos 2 (15%) foram realizados nos Estados Unidos; ademais, países como Arábia Saudita, Bélgica, Brasil, China, Egito, Espanha, França, Grécia, Itália, Londres e Suíça aparecem com apenas 1 (7,6%) estudo cada.

**Figura 2 -:** Diagrama de fluxo do processo de seleção dos artigos, PRISMA-ScR. São Carlos, SP, Brasil, 2023

**Figura 3 -:** Descrição dos artigos, segundo autor, ano, local, objetivo, tipo de estudo, amostra e principais resultados. São Carlos, SP, Brasil, 2023

**Autor, ano e local**	**Objetivo**	**Tipo de estudo e amostra (n)**	**Principais resultados**
Eita AAB, 2021, Estados Unidos da América^(7)^	Apresentar um caso da síndrome pós-aguda de COVID-19*, demonstrando a parostomia, disgeusia e língua gordurosa revestida de branco com papilas proeminentes	Estudo de caso. n = 1.	Após 14 dias da infecção por COVID-19*, paciente do sexo feminino, 31 anos, com queixa de disgeusia; apresentava língua branca gordurosa e desagradável, além de um olfato distorcido. Os sintomas melhoraram ao longo de quatro semanas com tratamento medicamentoso e após a eliminação viral.
Judant EM, 2021, França^(8)^	Determinar as características de pacientes com queixas respiratórias e as relações entre dispneia, anormalidades radiológicas e comprometimento funcional	Estudo de coorte. n = 478 (caso) e n =177 (controle).	Indivíduos hospitalizados por COVID-19* foram avaliados por consulta telefônica. A idade média da amostra foi de 61 anos e 42,1% era do sexo feminino. 4 meses após o diagnóstico da infecção viral, 78 pessoas relataram dispneia e 23 relataram tosse. Comparativamente aos indivíduos sem dispneia, os indivíduos com dispneia eram mais jovens (56,1±12,3 *versus* 61,9±16,6 anos, p^†^=0,001). Os indivíduos com dispneia apresentaram episódios iniciais mais graves de COVID-19*, com maior tempo de internação hospitalar [13 (7–23) *versus* 8 (4–14) dias, p<0,001] e internação mais frequente na Unidade de Terapia Intensiva (56,4 *versus* 24,5%, p<0,001).
Moreno-Perez O, 2021, Espanha^(9)^	Analisar a incidência da síndrome pós-aguda de COVID-19* e seus componentes e avaliar os fatores de risco associados à fase aguda da infecção	Estudo de coorte. n = 277.	Indivíduos com COVID-19* foram avaliados 77 dias após a infecção. A idade média foi de 62 anos e 52,7% do sexo masculino. Os sintomas pós- COVID-19* foram detectados em 50,9% dos pacientes. Os sintomas mais frequentes foram: dispneia e fadiga. Anosmia-disgeusia foi associada à idade mais jovem (<65 anos 24,9% (48/194) vs >65 anos 13,5% (11/83). Sintomas neurológicos relevantes (cefaleia, distúrbios de memória/deterioração cognitiva ou ambos) estavam presentes em 33 pacientes (11,9%). Após ajuste multivariado, nenhuma característica clínica basal, internação na unidade de terapia intensiva, tempo de permanência no hospital ou tratamento se comportam como preditores independentes de síndrome pós-COVID-19*.
Penetra SLS, 2021, Brasil^(2022)^	Descrever um caso de COVID-19* prolongado após infecção por SARS-CoV-2 Gama após um ano da primeira infecção em um profissional da saúde vacinado com CoronaVac no Brasil	Estudo de caso. n = 1.	Paciente do sexo masculino, 43 anos, sem comorbidades. Na infecção por COVID-19* apresentou febre, cefaleia, rinorreia e tosse seca; sintomas desapareceram em 14 dias. Nove meses após a infecção, recebeu a primeira dose da vacina e 28 dias após a segunda dose. Após 67 dias da segunda dose, desenvolveu um novo episódio de COVID-19* de linhagem Gama. Na ocasião, apresentou os mesmos sintomas; contudo, persistiram por 18 semanas após a infecção aguda com exceção da cefaleia e visão turva. Em ambas as infecções não houve necessidade de internação.
Tleyjeh IM, 2021, Arábia Saudita^(11)^	Avaliar a frequência de sintomas novos ou persistentes em pacientes com COVID-19* após a alta hospitalar e identificar fatores de risco associados	Estudo de coorte. n = 222.	Foram realizadas entrevistas telefônicas de acompanhamento com indivíduos com COVID-19* após uma mediana de 122 dias após a alta hospitalar. A maioria dos pacientes eram homens (77%) com idade média de 52,47 (± 13,95) anos. O tempo de diagnóstico dos sintomas pós-COVID-19* variou de 21 dias a 4 meses após a alta. 56,3% dos pacientes queixaram-se de persistência dos sintomas; 66 (29,7%) vivenciaram-nos por >21 dias e 64 (28,8%) relataram não ter retornado à saúde inicial. Falta de ar (40,1%), tosse (27,5%) e fadiga (29,7%) foram os sintomas mais frequentemente relatados. Após ajustes multivariados, o sexo feminino, a hipertensão pré-existente e o tempo de internação hospitalar foram associados a um risco aumentado de sintomas novos ou persistentes. A idade, a doença pulmonar pré-existente e as visitas aos serviços de urgência aumentaram a probabilidade de não recuperação total da COVID-19* aguda.
Tohamy D, 2021, Egito^(12)^	Avaliar as manifestações oculares da síndrome pós-aguda de COVID-19*	Estudo de coorte. n = 100 (caso) e n = 100 (controle).	O tempo de diagnóstico das condições pós-COVID-19* variou de 1 a 3 meses. Na amostra os indivíduos não tinham histórico de vacinação contra COVID-19*. A média de idade foi de 55,5 anos no grupo COVID versus 56,5 anos no grupo controle. No grupo COVID, 57% dos pacientes eram do sexo masculino versus 51% pacientes no grupo controle. No grupo COVID, 5 pacientes apresentavam oclusão vascular retiniana, 2 pacientes apresentavam neuropatia óptica isquêmica anterior, 3 pacientes apresentavam uveíte e 2 pacientes apresentavam serosa central. coriorretinopatia. Enquanto no grupo controle, 2 pacientes apresentavam oclusão vascular retiniana e nenhum apresentava, uveíte ou coriorretinopatia serosa central.
Carter SJ, 2022, Estados Unidos da América^(13)^	Descrever os efeitos concomitantes da infecção por SARS-CoV-2 quanto ao estado funcional, estado de humor e atividade física de lazer na síndrome pós-aguda de COVID-19*	Estudo caso-controle. n =17 (caso) e n =15 (controle).	O tempo de diagnóstico das condições pós-COVID-19* foi de aproximadamente 4 semanas. Incluídas 32 mulheres, sendo 17 diagnosticadas com COVID-19* e 15 controles. A pressão arterial diastólica (*p* = 0,031, ES = 0,80) e a RPP^‡^ ( *p*= 0,039, ES = 0,75) foram significativamente maiores entre os participantes do grupo COVID-19*. No grupo COVID-19*, observaram-se os seguintes sintomas: 41% com dores articulares/musculares por cerca de 300 dias, 53% com tosse no mesmo período; 29% com fadiga, 12% com comprometimento cognitivo e 71% com perda de paladar/cheiro no período aproximado de 100 dias, além de 35% com falta de ar.
Fanous J, 2022, Londres^(14)^	Avaliar objetivamente a fatigabilidade neuromuscular concomitante a fadiga e disfunção cognitiva relacionada à COVID-19* após 12 meses da infecção	Estudo de caso. n = 1.	O tempo de diagnóstico das condições pós-COVID-19* foi de cerca de 12 meses. Paciente sexo masculino, 27 anos, com queixa contínua pós-COVID-19* de fadiga e disfunção cognitiva, anosmia e ageusia , forte confusão mental, perda de apetite e insônia a mais de um ano após a infecção.
Jamoulle M, 2022, Bélgica^(1)^	Descrever de modo qualitativo e quantitativo o estado de saúde dos pacientes com Síndrome Pós-Aguda de COVID-19*	Estudo de coorte. n = 55.	O tempo de diagnóstico das condições pós-COVID-19* variou de 3 a 27 meses. 12% da população da amostra não foi vacinada durante o período do estudo. Dentre os pacientes, 72,7% eram mulheres, com idades compreendidas entre 12 e 79 anos. Após um ano de acompanhamento pode-se distinguir três tipos de evolução clínicas desde o início da COVID-19* aguda em 52 dos 55 pacientes: Grau 1 (leve) - duração de 3 a 8 meses, comprometimento respiratório, fadiga, dor esternal e exaustão com esforço (9 mulheres e 7 homens); Grau 2 (grave) - duração de 6 a 18 meses, fadiga extrema, exaustão ao esforço, anosmia, distúrbio cognitivo, distúrbio mnésico (12 mulheres e 3 homens); Grau 3 (muito grave) - duração de 12 a 27 meses, esgotamento constante, esforços impossíveis, revalidação cognitiva inútil, a hipersonia, o aumento do peso por inatividade, perturbações graves e persistentes a memória e ansiedade considerável. Outros sintomas variáveis citados nevoeiro cerebral com comprometimento da memória e déficit de recuperação de palavras, dispneia ao esforço e estado incapacitante.
Kim Y, 2022, Coréia do Sul^(15)^	Identificar sintomas prevalentes após 12 meses da infecção por COVID-19* em pacientes recuperados, fatores associados aos sintomas neurológicos e impacto a longo prazo da COVID-19 na qualidade de vida	Estudo de coorte. n = 900 (caso) e n = 241 (controle)	O tempo de diagnóstico das condições pós-COVID-19* variou de 6 a 12 meses. A idade média dos respondentes foi de 37 anos e 164 eram mulheres. Os sintomas persistentes por mais de 12 meses foram maiores naqueles classificados como gravidade moderada ou maior da doença durante a infecção aguda por COVID-19*. O sintoma mais frequente que durou até 12 meses foi dificuldade de concentração, observada em 54 pessoas, seguida por disfunção cognitiva (51), amnésia (48), depressão (43) e fadiga e ansiedade (39). Entre os sintomas persistentes relacionados com a COVID-19*, os problemas psiquiátricos tiveram um impacto maior na qualidade de vida do que quaisquer outros sintomas. Idade avançada, sexo feminino e gravidade da doença foram identificados como fatores de risco para sintomas neuropsiquiátricos persistentes.
Liu Q, 2022, China^(16)^	Investigar se a composição da microbiota intestinal está ligada à síndrome pós-aguda	Estudo de coorte. n = 106 (caso) e n = 68 (controle)	O tempo de diagnóstico das condições pós-COVID-19* variou de 3 a 6 meses.Os pacientes com agravamentos de COVID-19* foram acompanhados desde a admissão até seis meses. A idade média do grupo COVID foi de 48,3 anos e 52,9% eram do sexo feminino. Verificou-se as seguintes comorbidades: 17% hipertensos e 15,1% com diabetes mellitus. 81,1% dos pacientes relataram síndrome pós-aguda de COVID-19* em 3 meses. Os sintomas mais comuns foram: fadiga (31,3%), memória fraca (28,3%), queda de cabelo (21,7%), ansiedade (20,8%) e dificuldade em dormir (20,8%). Não houve diferenças significativas em idade, sexo, comorbidades, uso de medicamentos e gravidade da COVID-19* em pacientes com ou sem a síndrome pós-aguda de COVID-19*.
Mazzitelli M, 2022, Itália^(17)^	Avaliar a prevalência e os fatores associados à COVID-19* grave e a síndrome pós-aguda de COVID-19*	Estudo de coorte. n = 123.	O tempo de diagnóstico das condições pós-COVID-19* foi de 6 meses. Foram analisados pacientes diagnosticados com HIV^§^ e, concomitantemente, com COVID-19*. Dentre eles, 79,7% eram do sexo masculino, 77,2% eram de etnia caucasiana e a idade média foi de 51 anos. Nenhum dos pacientes havia sido vacinado para COVID-19*. Em relação às comorbidades: 30,9% eram hipertensos, 17,9% diabéticos e 15,4% tinham sobrepeso/obesidade; dentre os 75 pacientes que apresentaram infecção sintomática para COVID-19* e que sobreviveram, 26,7% dos pacientes relataram síndrome pós-aguda de COVID-19*. Quanto aos sintomas: 80% relataram astenia; 50% falta de ar relacionada aos esforços; 25% dor de cabeça recorrente; 15% queda de cabelo significativa; 10% perda do olfato; 0,5% diarreia crônica; além de 2 pacientes relatarem complicações cardiovasculares (infarto agudo do miocárdio e alterações rítmicas).
Zachariou A, 2022, Grécia^(18)^	Avaliar as consequências na função da bexiga em pacientes com síndrome pós-aguda de COVID-19* transferidos para a reabilitação de pacientes internados para cuidados prolongados após o tratamento inicial para a fisiopatologia de COVID-19*	Estudo descritivo. n = 147.	O tempo de diagnóstico das condições pós-COVID-19* variou de 4 a 7 semanas. Estudo realizado com pacientes com síndrome pós-aguda de COVID-19* encaminhados para o centro de reabilitação para cuidados prolongados. Desses, apenas 106 aceitaram a participar da pesquisa, sendo 45 (43,4%) do sexo feminino e 60 (56,6%) do sexo masculino. A idade média dos pacientes foi de 59,5. Identificou-se 75 pacientes com sintomas associados à Bexiga Hiperativa (BH) entre 4 a 7 semanas após apresentarem COVID-19*. Desses, 9 pacientes se recusaram a responder os questionários; 44 pacientes tiveram BH recém-diagnosticada e 22 pacientes com piora dos sintomas de BH. Todos os pacientes queixaram-se de noctúria.

*COVID-19 = *Corona Virus Disease 2019;* ^†^p = Nível de significância; ^‡^RPP = *Rate-Pressure Product*; ^§^HIV = *Human Immuno-Deficiency Virus*

Em relação aos dados demográficos, verificou-se que em cinco (38,4%) estudos a faixa etária média de casos de COVID Longa variou entre 42,4 e 55,5 anos, nos estudos em que a amostra foi composta por ambos os sexos, quatro descreveram suas amostras com os maiores percentuais correspondentes aos homens e em três estudos predominavam o sexo feminino.

Quanto aos sintomas mais frequentemente observados, destacou-se a fadiga (61,5%)^(1,7,9,11,13-16)^, dispneia (46,1%)^(1,8-9,11,13,17)^, alterações no olfato e/ou paladar (38,6)^(1,7,9,13,17)^, ansiedade (15,3%)^(1,15)^ e comprometimento cognitivo (30,7%)^(1,13-14,16)^.

Em relação às características clínicas quanto à presença de doenças preexistentes nas pessoas que manifestaram sinais e sintomas sugestivos de COVID Longa, apenas cinco (38,4%) estudos descreveram as comorbidades mais frequentes observadas em suas amostras, sendo predominante o relato de diabetes mellitus e hipertensão^(9,11,15-17)^.

Uma variável clínica pouco abordada nos estudos foi a relação do esquema vacinal contra COVID-19 e o desenvolvimento da COVID Longa. Verificou-se que nove (69,24%) estudos não mencionaram sobre a situação vacinal^(7-9,11,13-16,18)^, dois (15,3%) afirmaram que a população do estudo não foi vacinada^(12,17)^ e 1(7,6%) mostrou que aproximadamente 12% da população investigada não foi vacinada durante o período do estudo^(1)^. Nenhum dos estudos analisou a associação da vacinação com o desenvolvimento de sintomas da COVID Longa.

Outras lacunas identificadas nos estudos selecionados referem-se à ausência da análise da associação da COVID Longa às variáveis demográficas, como escolaridade e renda^(1,7-18)^ ou de variáveis clínicas sobre o histórico de hospitalização por COVID-19^(1,7,12-14,18)^.

No que se refere aos fatores de risco associados ao desenvolvimento da COVID Longa, apenas dois estudos descreveram uma associação positiva relacionada à gravidade na fase aguda na doença COVID-19, à presença de comorbidades, ao maior tempo de internação e internação em Unidade de Terapia Intensiva, à idade avançada e o sexo feminino ao risco aumentado de manifestar alguns sintomas da COVID Longa^(8,11,15)^. Por outro lado, um estudo apontou que a presença de anosmia-disgeusia foi associada à idade mais jovem (menores de 65 anos)^(9)^.

## Discussão

A partir da análise dos estudos selecionados nesta revisão, identificou-se uma ampla gama de sintomas relatados em pessoas que desenvolveram a doença COVID Longa, dentre os mais frequentementes estavam a fadiga, dispneia, alterações no olfato e paladar e distúrbios cognitivos relacionados à memória. Além disso, foi apontado que doenças como hipertensão arterial e diabetes mellitus foram comorbidades comumente encontradas nas pessoas. Nesta análise, observou-se uma maior prevalência de COVID Longa nas amostras em pessoas correspondentes à faixa etária pertencente aos adultos e em ambos os sexos. No entanto, alguns estudos associaram o sexo feminino como um fator de risco para o desenvolvimento da doença, outros fatores associados ao risco aumentado de desenvolver a COVID Longa foram relacionados à gravidade da infecção COVID-19, ao maior tempo de internação e à idade avançada. Ainda, em relação ao esquema vacinal contra a COVID-19, foi observada a ausência da abordagem dessa temática na maioria dos estudos analisados sobre a manifestação dos sintomas na doença COVID Longa.

Ressalta-se que no primeiro ano da pandemia, em 2020, o termo *“Long COVID”* foi utilizado para descrever os pacientes que se recuperaram da doença, mas que ainda apresentavam efeitos duradouros da infecção por mais tempo do que o esperado^(19)^. Ainda, o grau de infecção por COVID-19 pode estar relacionado a diferentes sintomas manifestados no desenvolvimento da COVID Longa. Assim, a COVID Longa foi observada com maior frequência nas pessoas que desenvolveram a forma grave da COVID-19 em comparação aos casos menos graves^(20)^. Ainda, o risco de desenvolver a COVID Longa aumenta com a idade, mas pode acometer toda a população, incluindo o público infantil^(21-22)^.

Embora a fisiopatologia da COVID Longa não esteja totalmente esclarecida, as hipóteses para a explicação da doença podem estar relacionadas à presença de reservatórios persistentes de SARS-CoV-2 em tecidos, desregulação imunológica, impactos do vírus na microbiota, autoimunidade, preparação do sistema imunológico a partir do mimetismo molecular, coagulação sanguínea microvascular com disfunção endotelial e sinalização disfuncional no tronco cerebral e/ou nervo vago^(22-23)^.

Em relação aos sintomas manifestados na COVID Longa, tem sido descrito que podem estar sobrepostos a diversas condições que não estejam relacionadas à própria infecção de COVID-19, como, por exemplo, uma síndrome pós cuidados intensivos nos casos que necessitaram de internação ou até mesmo a exacerbação de condições de saúde preexistentes^(24-25)^.

Nesse contexto, os sintomas manifestados na COVID Longa podem ter etiologias distintas. Sendo assim, na abordagem dos sintomas respiratórios de longo prazo, estes podem ser causados por distúrbios vasculares pulmonares resultantes do dano vascular pulmonar nos microvasos sofridos na infecção aguda do vírus SARS-CoV-2. Um dos sintomas mais relatados, a dispneia, na ausência de lesão pulmonar, poderia estar relacionada à regulação inadequada da ventilação resultante de distúrbios do sistema nervoso autônomo, com potenciais danos nos receptores reflexos intratorácicos ou no tronco cerebral/zonas corticais cerebrais^(23,26)^. Já no que se refere à manifestação de dores, incluindo dores articulares e musculares, elas poderiam ser atribuídas a mecanismos tromboinflamatórios em relação a lesões teciduais e processos autoimunes^(27-28)^.

Quanto aos sintomas neurológicos apresentados na COVID Longa, uma análise apontou que sequelas persistentes poderiam estar relacionadas a complicações neurológicas agudas na infecção pelo vírus SARS-CoV-2, resultantes de danos cerebrais ou de outros fatores relacionados à hospitalização. Dessa maneira, a manifestação dos distúrbios cognitivos, cefaleia e alterações olfativa e/ou gustativa poderiam depender de uma fisiopatologia independente da fase aguda. Uma explicação seria que a propagação do vírus SARS-CoV-2 no cérebro, por meio da cavidade nasal ou da corrente sanguínea, resultaria em uma neuroinflamação, e esta, sendo persistente, poderia ser responsável por comprometimento neurocognitivo ou distúrbios de saúde mental^(23)^.

Desse modo, exames clínicos de pessoas com COVID Longa apresentam uma diminuição de serotonina causada pela infecção de COVID-19 devido à redução da absorção de triptofano, trombocitopenia e aumento da expressão da flavoenzima monoaminoxidase, que resulta na diminuição da ativação vagal e hipocampal e, também, no considerável comprometimento cognitivo. Ainda, foi constatado que a redução da serotonina e a disfunção do nervo vago podem ser associadas à COVID Longa, pois são sugestivas de um trajeto posterior à infecção viral^(29)^.

Contudo, a partir da ideia de que a fenotipagem metabolômica, proteômica e imunológica de pacientes que contraíram o SARS-CoV-2 combinou com uma diversidade de sintomas clínicos com potenciais biomarcadores para a COVID-19, o metabolismo desregulado e a inflamação podem contribuir para os sintomas da COVID Longa. Nesse contexto, destaca-se que os triglicerídeos sanguíneos totais e os metabólitos lactato e piruvato foram mais elevados e as lipoproteínas foram mais baixas em pacientes com COVID Longa quando comparado com os controles saudáveis^(30)^.

Outrossim, foi possível verificar que as citocinas ou o sexo feminino e/ou masculino estão relacionados com os metabólitos, como citrato, glutamato e histidina, em pacientes que apresentaram os sintomas como fadiga crônica, dispneia e confusão mental no curso longo da doença. Enfim, várias citocinas e quimiocinas foram correlacionadas com metabólitos e lipoproteínas, sendo a desregulação no metabolismo e a inflamação consideradas como potenciais fatores para o desenvolvimento de COVID Longa^(30)^.

Dentre os fatores de risco para o desenvolvimento da COVID Longa, foram descritos a hospitalização em decorrência da infecção, o aumento da idade, ser do sexo feminino e portador de comorbidades^(31)^. Nesta conjuntura, sabe-se que doenças comumente encontradas na população, como diabetes mellitus quando mal controladas causam danos aos órgãos, ocasionando, especificamente, lesões microvasculares que podem ser exacerbadas na infecção por SARS-CoV-2 em sua forma grave. Além disso, o estado inflamatório pré-existente pode se manter exacerbado e agravado após a infecção^(32)^.

As síndromes coronarianas agudas, insuficiência cardíaca, arritmias, acidente vascular cerebral e tromboembolismo também perduram além do estágio inicial da infecção por SARS-CoV-2, prolongando-se por vários meses. Sintomas cardiopulmonares como dor no peito, falta de ar, fadiga e taquicardia ortostática postural podem ser comuns na COVID Longa e associados à incapacidade significativa e ao aumento da ansiedade. Contudo, os mecanismos fisiopatológicos para as complicações cardiovasculares tardias não estão bem compreendidos^(33)^.

A literatura aponta divergências em relação à prevalência de COVID Longa. Um estudo que analisou a população do norte dos Países Baixos descreveu que a doença pode ocorrer em cerca de uma a cada oito pessoas com a doença na população geral, enquanto outra pesquisa na Itália relatou que nove em cada dez pacientes, após se recuperarem da doença, ainda apresentavam pelo menos um sintoma depois de 60 dias do início^(19,34)^.

Além disso, a prevalência de COVID Longa difere entre homens e mulheres, considerando as diferenças fisiológicas e hormonais que influenciam no sistema imunológico. Dessa forma, as mulheres possuem níveis mais elevados de imunoglobulina, fazendo com que desenvolvam respostas imunológicas mais fortes após a imunização ou infecção quando comparadas aos homens, ao passo em que apresentam maior suscetibilidade às doenças autoimunes^(35)^.

Assim, as mulheres podem apresentar diferentes fatores de risco para a COVID Longa a depender do estado hormonal. Nesse contexto, a menopausa é um fenômeno que está associado a um risco aumentado para a doença, sendo que mulheres na pós-menopausa apresentaram maior incidência de COVID-19 quando comparadas com mulheres na pré-menopausa. Desse modo, é sugestivo que o estrogênio possa estar associado à gravidade da doença; também, destaca-se que a ECA2 é encontrada no cromossomo X, sendo o estrogênio um regulador negativo da expressão de ECA2. Ainda, o receptor *Toll-like* 7 (TLR7), regulador da produção de interferon (IFN), é considerado um gene imune do cromossomo X que ocasiona maior sinalização de IFN na COVID-19 e melhor depuração viral em mulheres, contudo, a sinalização contínua as predispõe a um maior risco de desenvolver um quadro de COVID Longa^(36)^.

Entretanto, em ambos os sexos, nos achados clínicos, as alterações nas células T, o número reduzido de células de memória efetoras *cluster of differention* 4+ (CD4+) e *cluster of differention* 8+ (CD8+) e a expressão elevada da proteína PD1 em células de memória central persistiram por pelo menos 13 meses em indivíduos com COVID Longa que tiveram COVID-19 leve^(22,37)^.

Em indivíduos que desenvolveram a COVID Longa, detectou-se uma diminuição de imunoglobulina M (IgM) desde a infecção primária até o acompanhamento de seis meses. Ainda, enquanto a imunoglobulina G1 (IgG1) ficou inalterada, a imunoglobulina G3 (IgG3) foi menor em pacientes com COVID Longa, ocorrendo o contrário nas concentrações de IgG3 em casos leves e graves da COVID-19. Dessa forma, foi possível observar uma assinatura da imunoglobulina que, combinada com a idade, história de asma brônquica e cinco sintomas durante a infecção primária, pode prever o risco de COVID Longa independentemente do período em que foi realizada a coleta de sangue^(38)^.

Por meio de títulos de imunoglobulina G (IgG) específicos para SARS-CoV-2 distribuídos igualmente por um período conforme a idade, sexo e ausência/presença de sintomas longas, os pacientes foram vacinados entre o 10º e 15º meses após a infecção, identificando que a administração da vacinação precoce é mais eficaz que a tardia. Ainda, a vacinação administrada antes das infecções por SARS-CoV-2 está associada com uma menor prevalência dos sintomas persistentes^(39)^.

Nesse contexto, embora a vacinação contra a COVID-19 tenha sido uma variável clínica não analisada nos estudos selecionados nesta revisão, a sua relação com a COVID Longa merece destaque. A literatura aponta que a vacinação dupla culminou em efeito benéfico temporário nos sintomas longos com duração de 21 a 67 dias^(39)^. Em uma revisão sistemática que analisou dados sobre a vacinação antes e após a infecção pelo vírus SARS-CoV-2, em relação à vacinação contra a COVID-19 antes da infecção, mostrou-se que os indivíduos apresentaram uma redução significativa na incidência de COVID Longa. Por outro lado, ao se analisar a remissão e a recuperação da COVID Longa, há chances de não recuperação quando os pacientes foram vacinados após a infecção^(40)^.

Desse modo, a vacinação contra a COVID-19, além de atuar na diminuição da gravidade da doença e nas taxas de hospitalização, tem sido associada a uma considerável redução no desenvolvimento de sintomas da COVID Longa^(40-41)^. Nessa perspectiva, é notória a importância da adesão à vacinação contra a COVID-19 uma vez que, sendo reconhecida, atua como instrumento de proteção para o desenvolvimento do longo curso da doença, visto que ainda não existem tratamentos amplamente eficazes tanto para a doença aguda como COVID Longa^(22)^.

Por fim, é importante destacar que os cuidados à saúde direcionados às pessoas com COVID Longa podem estar comprometidos, especialmente em países de baixa e média renda, devido aos sistemas de saúde limitados e com poucos recursos, podendo se apresentar como uma grande demanda aos sistemas de saúde já sobrecarregados, exacerbando ainda mais as desigualdades em saúde^(42)^.

Esta revisão de escopo contribui na identificação dos sintomas mais frequentes manifestados na COVID Longa, dentre eles fadiga, dispneia, alterações no olfato e paladar e distúrbios cognitivos relacionados à memória. Além disso, foi observada nos estudos selecionados uma maior prevalência de COVID Longa em pessoas correspondentes à faixa etária pertencente aos adultos e em ambos os sexos.

Constituem limitações do estudo a inclusão somente de estudos em português, inglês e espanhol, de artigos que se encontravam disponíveis em texto na íntegra, além das possibilidades contidas nas bases de indexação não incluídas nesta pesquisa.

## Conclusão

O mapeamento da literatura evidenciou a diversidade de sintomas em pessoas que desenvolveram COVID Longa, sendo mais prevalentes em pessoas adultas, de ambos os sexos e com a presença de comorbidades. Devido à elevada carga de sintomas persistentes ocasionados após a infecção pelo SARS-CoV-2, a COVID Longa é um desafio para os serviços de saúde, principalmente no que tange à diversidade de manifestações sintomáticas. Tal situação implica em dificuldades para a qualidade de vida das pessoas acometidas pela COVID Longa.

Esta revisão encontrou importantes lacunas que podem influenciar na compreensão da COVID Longa, desse modo, recomenda-se realizar estudos futuros sobre a abordagem da gravidade e persistência dos sintomas apresentados na COVID Longa e sobre a relação da adesão ao esquema vacinal contra a COVID-19, interseccionados pelos aspectos de sexo, raça/etnia, grau de susceptibilidade nas diferentes faixas etárias, nível de escolaridade e renda, além das comorbidades mais recorrentes na população. O aprofundamento de estudos utilizando essas variáveis pode contribuir para a efetividade de estratégias de saúde pública para o diagnóstico e assistência às pessoas acometidas pela doença.
